# Nanoscopic Mapping of the Extracellular Space in Amyloid Plaque‐rich Cortex

**DOI:** 10.1002/advs.202515674

**Published:** 2025-10-24

**Authors:** Juan Estaún‐Panzano, Yulia Dembitskaya, Ivo Calaresu, Somen Nandi, Quentin Gresil, Evelyne Doudnikoff, Thierry Leste‐Lasserre, Thierry Amédée, Laurent Cognet, Laurent Groc, Urs Valentin Nägerl, Erwan Bezard

**Affiliations:** ^1^ Institut des Maladies Neurodégénératives CNRS Université de Bordeaux UMR 5293 Bordeaux F‐33000 France; ^2^ Institut Interdisciplinaire de Neurosciences CNRS Université de Bordeaux UMR 5297 Bordeaux F‐33000 France; ^3^ Laboratoire Photonique Numérique et Nanosciences CNRS Université de Bordeaux UMR 5298 Talence F‐33400 France; ^4^ Laboratoire Photonique Numérique et Nanosciences IOGS CNRS UMR 5298 Talence F‐33400 France; ^5^ Neurocentre Magendie INSERM Université de Bordeaux U1215 Bordeaux 33000 France; ^6^ Institut für Anatomie und Zellbiologie Universitätsmedizin Göttingen Georg‐August‐Universität 37075 Göttingen Germany

**Keywords:** Alzheimer's disease, amyloid plaques, diffusion, extracellular space, single particle tracking

## Abstract

A hallmark of Alzheimer's disease (AD) is the accumulation of amyloid plaques, primarily composed of misfolded amyloid β (Aβ) peptides. Complementary high‐resolution imaging techniques are employed to investigate the plaque penetrability and the extracellular space (ECS) rheology in a mouse model of AD. Two‐photon shadow imaging in vivo confirms that a dense ring of cells surrounds cortical amyloid plaques but highlights the diffusional penetrability of the amyloid core. Quantum dot tracking unveils that ECS diffusional parameters are heterogeneous in and around plaques, with an elevated diffusivity within and around plaques compared to wild‐type‐tissue. The amyloid core shows low nanoparticle density, varying by plaque phenotype. Carbon nanotube tracking confirms these altered local rheological properties at the level of the whole cortex of AD mice. Finally, the extracellular matrix is found to be dysregulated within the amyloid plaque, which may account for the observed alterations in diffusivity. This study provides fresh insights for understanding Aβ plaque penetration, a prerequisite for therapeutic development.

## Graphical abstract

The extracellular space and diffusion around amyloid plaques are examined using shadow imaging and single‐particle tracking. Increased diffusivity is found near plaques, extracellular matrix alterations, and plaque core penetrability that varies with amyloid phenotype.

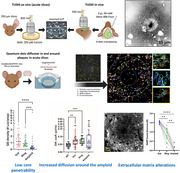

## 1 Introduction

Alzheimer's disease (AD) poses significant challenges to researchers and clinicians due to its poorly understood etiology and pathophysiology. This condition profoundly affects cognitive function, especially in the expanding aging population, becoming a significant socioeconomic burden for healthcare systems.^[^ ^1^ ^]^ A recognized hallmark of the disease is the presence of amyloid plaques, primarily composed of misfolded amyloid β (Aβ) peptides, in the brain's extracellular space (ECS).^[^ ^2^, ^3^ ^]^

Among recent strategies to tackle the disease, amyloid plaque clearance and immune system modulation have raised hopes but still suffer from unsatisfactory outcomes.^[^ ^4^ ^]^ As a consequence, the penetrability of brain tissue has come into focus as a potentially pivotal determinant of therapeutic efficacy.^[^ ^5^ ^]^ However, how different substances can navigate within the ECS remains poorly understood, let alone whether they can penetrate amyloid plaques. Deeper insights into the properties of the ECS are crucial, as it plays a key role in normal brain function^[^ ^6^ ^]^ and disease progression.^[^ ^7^, ^8^, ^9^ ^]^

This study presents an innovative experimental and analytical framework for dissecting the influence of the ECS rheology, i.e., a concept that encompasses diffusionability within the compartment and compartment dimensions, on molecular diffusion in the context of amyloid pathology. We used 2‐photon shadow imaging and nanoscopic single‐particle tracking (SPT) imaging to investigate the morphology and diffusional properties of the ECS, i.e., its rheology, in the transgenic amyloid precursor protein (APP)/PS1 mouse model of AD. Our study provides new insights into the factors that influence the penetration of exogenous molecules into Aβ plaques themselves and Aβ‐plaques‐bearing cortex.

## 2 Results

### 2.1 Shadow Imaging Reveals Amyloid Plaque Organization and Surrounding ECS Structure

**Figure 1:**
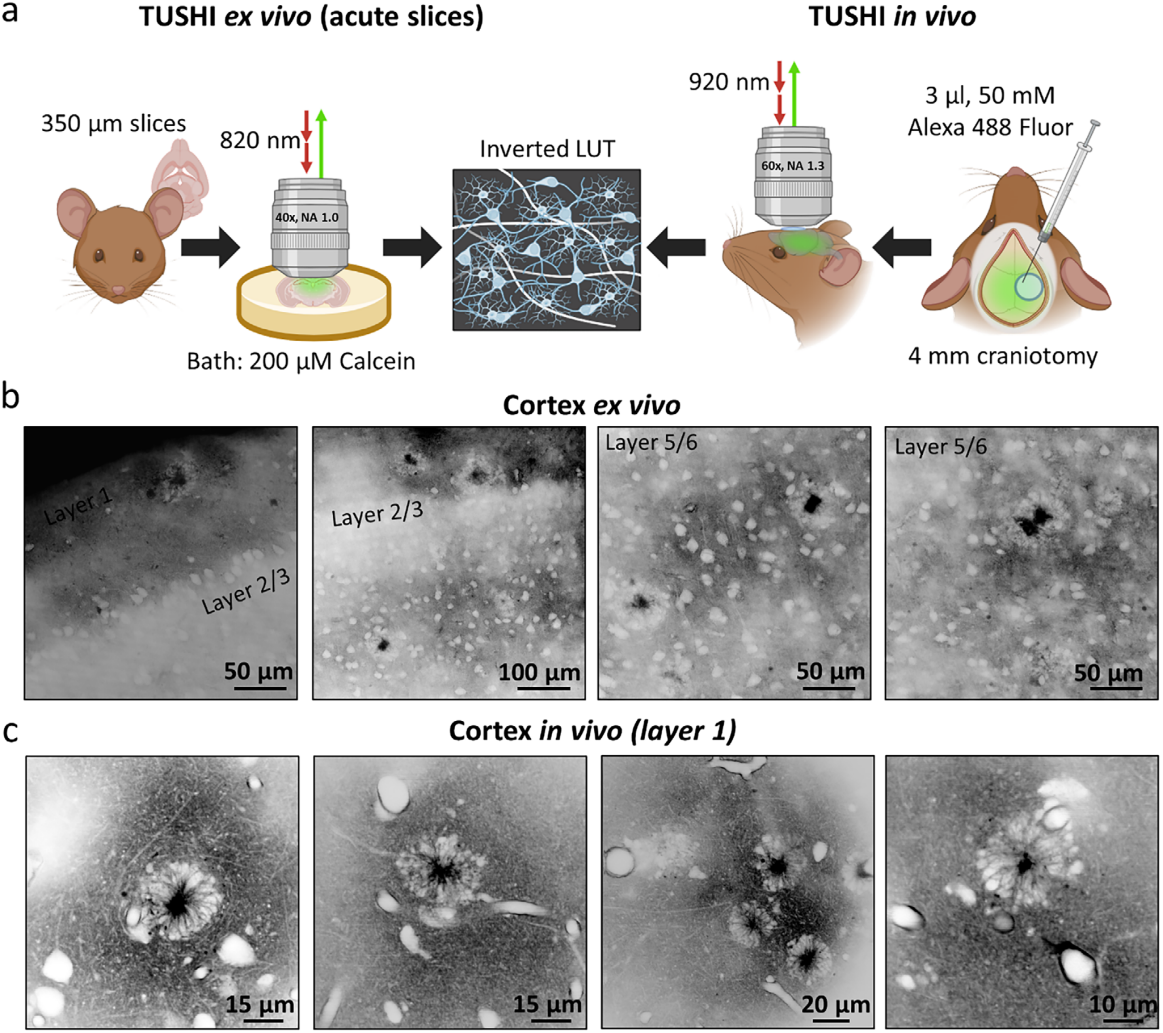
Visualization of amyloid plaques using TUSHI in the cortex ex vivo and in vivo. a) Schematic representation of the visualization of amyloid plaques using TUSHI in vitro and in vivo. TUSHI ex vivo: acute brain slices (350 µm thick) were placed into a microscope chamber after incubation and 200 µm of Calcein was bath applied to label the extracellular space. TUSHI in vivo: Alexa 488 Fluor (3 µL, 50 mm) was injected in the lateral ventricle on the side of 4 mm craniotomy and covered with a glass coverslip afterward. All collected images are presented in an inverted LUT where extracellular space appears black and brain structures–white. b) Representative images of amyloid plaques in the cortex ex vivo. c) Representative images of amyloid plaques in the cortex in vivo. Created in BioRender. Bezard, E. (2024) BioRender.com/i34k786.

We focused our study on the sensory and motor cortices. The amyloid pathology burden starts as early as 3 months of age and displays a significant load of plaques in 8 months old female APP/PS1 mice.^[^ ^10^ ^]^ To visualize amyloid plaques in their native environment, we used 2‐photon shadow imaging (TUSHI) ex vivo in acute brain slices and in vivo in anesthetized mice, achieving sub‐micrometer resolution and panoptical visualization of brain tissue architecture.^[^ ^11^ ^]^ We labeled the ECS using cell‐membrane‐impermeable dyes (Calcein bath applied in acute brain slices, and Alexa Fluor 488 injected intracerebroventricularly in vivo, see the Experimental Section for details), projecting all luminal structures (cells, blood vessels) as black “shadows” in the images. For better illustration, we inverted the image contrast so that all cellular structures appear white and the ECS black (**Figure** **1**a). In acute brain slices, the approach detected amyloid plaques in the cortex (Video S1, Supporting Information). Their amyloid region was filled with Calcein (black in the inverted images). The ex vivo finding was confirmed in vivo after intracerebroventricular injection of Alexa 488 Fluor (Videos S2,S3, Supporting Information). Notably, amyloid plaques were frequently associated with blood vessels (Videos S2,S3, Supporting Information), which support the notion of neurovascular alterations in AD pathology.^[^ ^12^, ^13^ ^]^ Both dyes appeared uniformly distributed in the center of the amyloid plaques, but this does not mean that the ECS in this region is empty (it is chock‐full with proteins, lipids, organelles, and dystrophic neurites with compromised membranes).^[^ ^14^, ^15^ ^]^ Instead, it shows that small organic dyes with hydrodynamic diameters on the order of a nanometre have no trouble penetrating amyloid plaques, which have an internal structure that cannot be resolved by diffraction‐limited light microscopy.

Plaques were irregularly distributed in the brain parenchyma and present in all cell layers of the cortex (layers 1–6 (Figure 1b). Notably, they always featured a dense sphere of cells that looked like a ring in optical sections (Videos S1–S3, Supporting Information), surrounding the core of the amyloid plaque, as previously reported in many instances.

We then performed TUSHI through a cranial window in the somatosensory cortex in vivo (Figure 1c), which confirmed the ring/amyloid arrangement as a consistent feature in the diseased brain (Videos S2, S3, Supporting Information), which was often associated with blood vessels (Figure 1c). By contrast, we never observed such structures in age‐matched wild‐type (WT) mice (Figure S1 and Videos S4,S5, Supporting Information).

**Figure 2:**
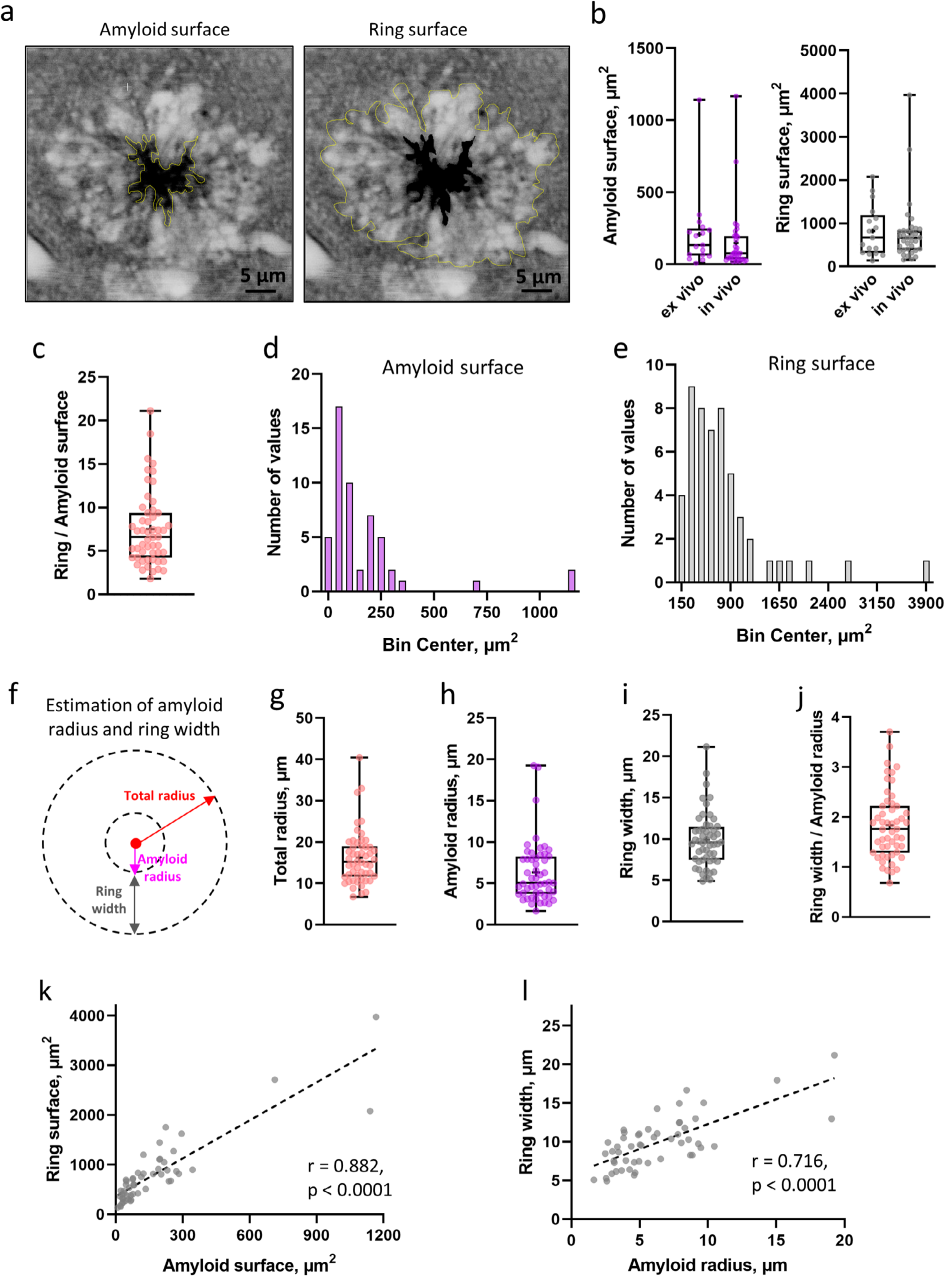
Dimensions of amyloid plaques using TUSHI. a) Manually selected ROIs were calculated using the “free‐shape” drawing tool in ImageJ, from which the amyloid and ring surface area were calculated. b) Left panel–amyloid surface in cortex ex vivo (134.2 µm^2^, IQR = 61.63–248.7, *n* = 17) and in vivo (77.0 µm^2^, IQR = 35.6–195.6, *n* = 35) (Mann–Whitney test, *p* = 0.134), right panel–ring surface in cortex ex vivo (666.7 µm^2^, IQR = 312.3–1193, *n* = 17) and in vivo (666.4 µm^2^, IQR = 381.5–833.1, *n* = 35) (Mann–Whitney test, *p* = 0.671). c) The surface of the ring was 6.6 times higher than that of amyloid plaque in the cortex (ex vivo and in vivo) (IQR = 4.24–9.36, *n* = 52). d) Log‐normal distribution of amyloid surface in the cortex (ex vivo and in vivo) (D'Agostino and Pearson test, *p* = 0.765, *n* = 52). e) Log‐normal distribution of ring surface area in the cortex (ex vivo and in vivo) (D'Agostino and Pearson test, *p* = 0.910, *n* = 52). f) Approximation of amyloid plaques as a circle to calculate amyloid radius and width of the ring. The total radius (amyloid + ring) was calculated from the surface of the amyloid + ring. The width of the ring was calculated as the difference between the total radius and the radius of the amyloid. g) Estimation of total radius of amyloid + ring in cortex (ex vivo and in vivo) (15.21 µm, IQR = 11.62–18.98, *n* = 52). h) Estimated amyloid radius in cortex (ex vivo and in vivo) (5.1 µm, IQR = 3.71–8.25, *n* = 52). i) Estimated width of the ring in the cortex (ex vivo and in vivo) (9.57 µm, IQR = 7.47–11.44, *n* = 52). The width of the ring was 1.82 times higher than that of the amyloid radius in the cortex (ex vivo and in vivo) (IQR = 1.29–2.22, *n* = 52)). k) Positive correlation of ring and amyloid surface (Pearson's *r* = 0.882, *p* < 0.0001). l) Positive correlation of ring width and amyloid radius (Pearson's *r* = 0.716, *p* < 0.0001). All data are represented as median and IQR, minimum, maximum and mean (labeled as a cross) with individual data points.

We measured the surface area of the amyloid cores and the rings around them ex vivo and in vivo in the central image section of a plaque (**Figure** **2**a). The surface areas of the amyloid cores and cell rings were indistinguishable between the ex vivo and in vivo conditions (Figure 2b). The surface area of the cell ring was about 6.6 times bigger on average than the surface area of the core (Figure 2c). The surface areas of all measured amyloid cores (Figure 2d) and their cell rings (Figure 2e) follow a log‐normal distribution, consistent with X‐ray phase‐based virtual histology.^[^ ^16^ ^]^

Approximating a spherical geometry for the plaques comprised of amyloid core and cell ring, we calculated their radius and width, respectively (Figure 2f). The amyloid radius was 5.1 µm (Figure 2h), while the ring width was 9.57 µm (Figure 2i), making the width 1.82 times bigger than the radius (Figure 2k). The median radius of amyloid plaques (amyloid core + cell ring) was 15.2 µm (Figure 1g). The radius of the amyloid core and the width of the ring were indistinguishable between the ex vivo and in vivo conditions (Figure S2, Supporting Information). Finally, we observed a positive correlation between the surface areas of the amyloid core and cell ring (Figure 2l). Likewise, radius and width were positively correlated (Figure 2m), meaning larger amyloid cores had wider cell rings. Remarkably, these numbers and geometrical assumptions indicate that, on average, the entire plaque structure takes up about 26 times more volume than the amyloid core by itself.

The strength of our novel approach lies in clearly distinguishing between membrane‐compromised structures (which the dye penetrates) and intact ones, while also providing precise size measurements of the different regions of the plaque (core vs ring). While TUSHI does not provide diffusional information, it allows us to identify regions of interest (ROIs) for further blind quantum dot (QD) SPT analysis.

### 2.2 Probing Amyloid Plaque Accessibility by Quantum Dot Tracking

TUSHI revealed that plaques come with a dense ring of cells, which might affect molecular access to the central core of the amyloid plaque. To study the accessibility of the amyloid plaques to macromolecules, we deployed photoluminescent QDs emitting at a wavelength of 655 nm. These particles are passivated by a polyethylene glycol (PEG) layer, and decorated with F(ab')2 IgG (H+L, goat anti‐rabbit) fragments to confer antibody‐like diffusive behavior.^[^ ^17^ ^]^ Moreover, their diameter is comparable to biological macromolecules (e.g., immunoglobulins, 10–20 nm) and nanocarriers (e.g., exosomes, 30–150 nm) present in the cerebrospinal fluid (CSF).^[^ ^18^, ^19^ ^]^ We characterized the probes by imaging the dry size of the QD core and its organic shell (≈25 nm) using transmission electron microscopy of negatively stained nanoparticles (Figure S3a, Supporting Information). Additionally, we determined their hydrodynamic diameter (33.6 ± 1.2 nm) in physiological saline using dynamic light scattering (DLS) (Figure S3b, Supporting Information).

**Figure 3:**
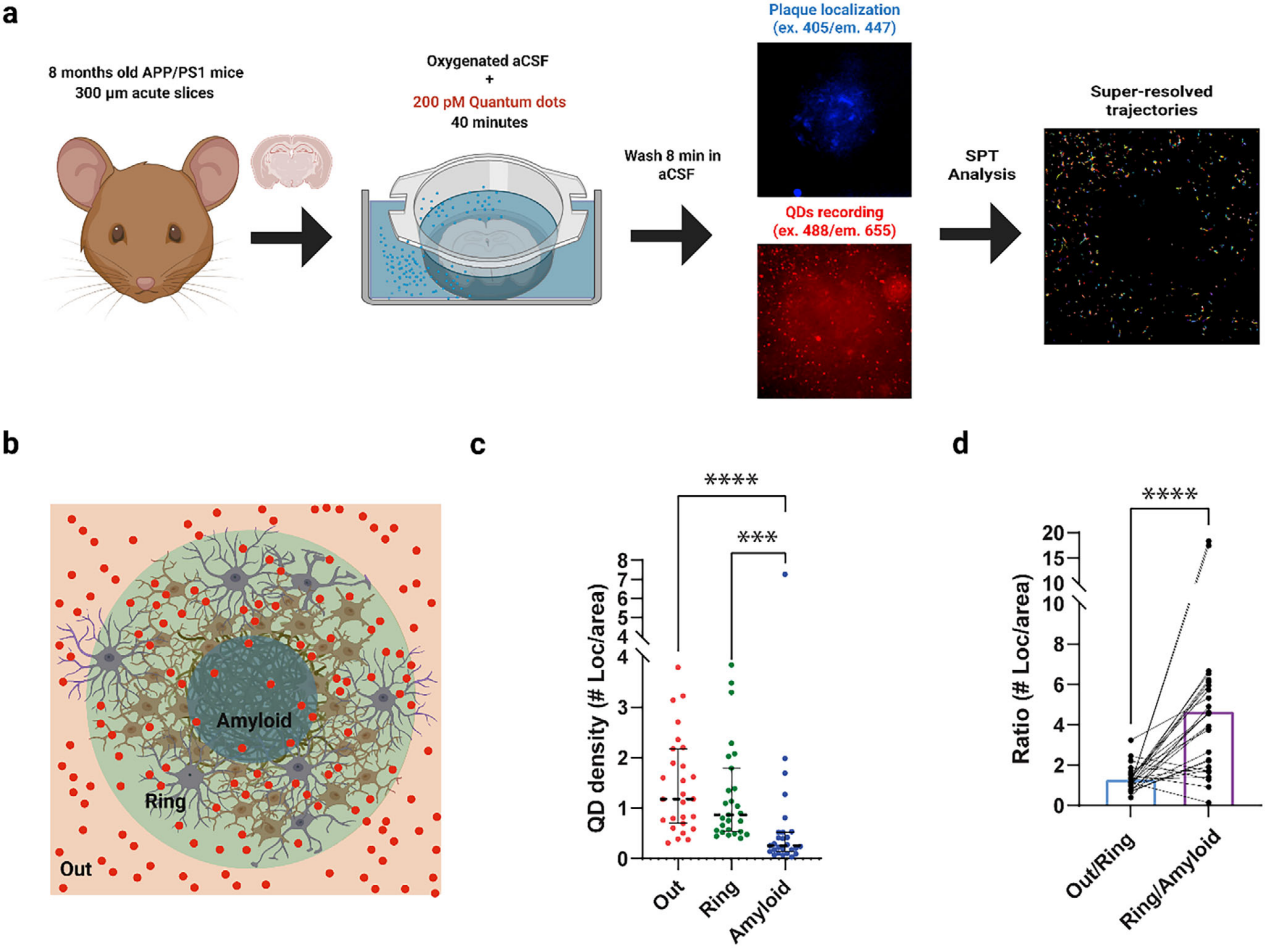
QD video‐tracking enables the investigation of nanoscale diffusion dynamics within amyloid plaques. a) Schematic workflow of the QDs tracking in acute slices. 8 months old females are sacrificed by cervical dislocation, and acute brain slices are then prepared in a bubbled ice‐cold NMDG‐based solution. After recovery, slices are incubated for 40 min in oxygenated warm aCSF containing QDs (about 200 pm) and washed another 8 min in aCSF only. Slices are imaged in an upright microscope while perfused with aCSF at 35°C under constant carbogen bubbling. Plaque was located using 405 excitation autofluorescence. QDs were imaged using 488 excitations and an emission filter. Analysis gauges the building of super‐resolved trajectories of particles (see the Experimental Section and Figure S6 in the Supporting Information for further information). b) Sketch of identified anatomical compartments in the proximity of senile plaques, consisting of 3 concentric ROIs: we determine QD (sparse red dots) behavior as they move from the cortical parenchyma (Out), through cell dense envelope (Ring), until the center of Aβ plaques (Amyloid). c) We found QD localizations were scarcer inside the amyloid ROI in a plaque‐dependent manner. To quantify this effect, we have quantified the particle density by ROI quantifying superlocalizations per unit area (Out: Median = 1.18, IQR = 0.69–2.1, Ring: Median = 0.88, IQR = 0.54–1.79, Amyloid: Median = 0.26, IQR = 0.14–0.52, Friedman test multiple comparisons: Out vs Ring *p*‐value=0.17, Out vs Amyloid *p*‐value < 0.0001, Ring vs Amyloid *p*‐value = 0.0002). *N*_APP/PS1_ = 27 plaques, from 5 mice. The graph shows median and IQR. d) We defined a penetrability ratio by dividing the number of QD points in the outside ROI per area by the number of QD points inside the ring ROI per area. Higher ratio values mean that QDs cannot access the inner structure, while values close to 1 mean an even distribution of points. The Outside/Ring ratio gauges a median value of median = 1.14, IQR = 0.98–1.51, pointing out that the ring itself does not importantly impede the access to diffusing molecules, while Ring/Amyloid shows a fourfold higher ratio of median = 3.97, IQR 1.75–5.96 (Wilcoxon test *p*‐value < 0.0001). *N*_APP/PS1_ = 27 plaques, from 5 mice. Created in BioRender. Bezard, E. (2024) BioRender.com/w96n099.

Incubating acute brain slices for 40 min, QDs penetrate the tissue and navigate the ECS (Videos S6,S7, Supporting Information, respectively, APP/PS1 and WT cortex). Their dimensions, isotropy, and photostability allow tracing their trajectories as they diffuse in the ECS. To locate amyloid plaques within the cortex in ex vivo carbogenated tissue (**Figure** **3**a), we exploited the characteristic autofluorescence of plaque cores under confocal excitation with 405 nm light (Figures S4,S5, Supporting Information).^[^ ^20^ ^]^ This allowed us to draw a concentric ROI around autofluorescent amyloid cores and to superlocalize QDs (Figure S6, Supporting Information) inside the plaque, quantifying their accessibility and mobility in the regions of the amyloid core (Amyloid), cellular ring (Ring), and the immediate surrounding tissue (Out) (Figure 3b). While the cell ring was not visualized in the SPT experiments, we placed the ROI for it according to the TUSHI measurements, extending the radius of the amyloid core by a factor of 1.82 (Figure S7, Supporting Information).

We measured QD densities in the three compartments for all recorded fields (Figure 3c). The density of QDs was the highest in the surrounding tissue, intermediate in the cell ring, and the lowest inside the amyloid core (Figure 3c). To assess potential changes in particle behavior between the different compartments, i.e., to define whether a specific interface is preventing penetration, we defined two interfaces (Out–Ring and Ring–Amyloid) between pairs of adjacent ROIs. We calculated the QD density ratio for these pairs. Localization density ratios between the surrounding cortical tissue and the cell‐rich ring distributed around 1 with low variability (Figure 3d), indicating that the interface between cortical neuropil and the cell ring does not appear to impose a barrier for molecular diffusion. Ratios of QD density within the ring versus the amyloid core were characterized by a fourfold increase, indicating a low penetrability and/or retention by the amyloid core (Figure 3d).

This analysis indicates that i) QDs penetrate the cell ring and the core of the amyloid plaques, but ii) the overall plaque structure restricts macromolecule availability within the amyloid. We next moved to clarify how macromolecule availability might be affected by plaque diversity.

### 2.3 Different Amyloid Cores Exhibit Distinct Penetrability

Extracellular amyloid in plaques exists in many shapes and sizes that could be related to multiple mechanisms of formation and toxicity.^[^ ^21^ ^]^ Amyloid pathology is well characterized throughout AD progression, but the functional impact, temporal progression, and neurotoxic effects of various types of Aβ deposits are still not fully understood.^[^ ^22^ ^]^ Some authors have suggested that microglial processes in early filamentous plaques envelop individual fibrils, facilitating their compaction. Dense core plaques, in turn, can be considered as more mature and less toxic forms.^[^ ^15^, ^22^, ^23^ ^]^ Approaching amyloid plaque diversity from an extracellular perspective may offer insights into plaque‐specific properties and their implications for disease progression and treatment.

**Figure 4:**
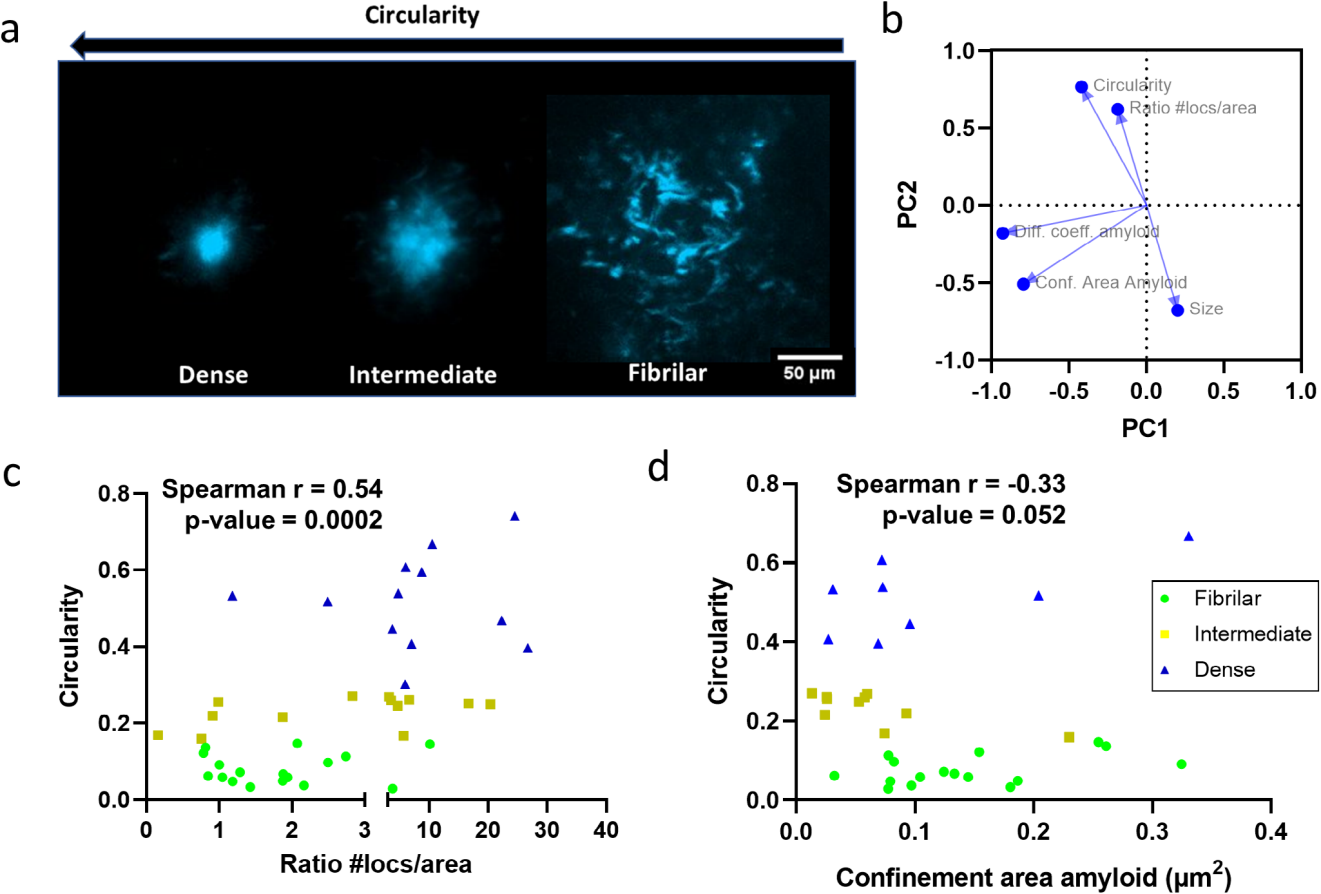
QD penetration depends on plaque core phenotype. a) After QD recording, each plaque ThS signal was associated with a circularity value as a proxy of plaque phenotype (see the Experimental Section). b) Principal component analysis (PCA) loadings plot shows association between circularity and QD density ring/amyloid ratio while suggesting a lack of linear correlation with rheological parameters (PC1 total variance = 34.9%, eigenvalue = 1.746:PC2 total variance = 34.4%, eigenvalue = 1.720). c) Scatter plot of Circularity versus ratio #locs/area (amyloid impenetrability) shows that as plaque densifies, penetration becomes more difficult. The relationship correlates significantly (Spearman *r* = 0.54, *p* = 0.0002). Amyloids are categorized as previously defined^[^ ^15^ ^]^ (fibrillar = 0.00–0.14, intermediate 0.15–0.28, compact >0.28). d) We observed a strong trend for negative correlation between circularity and confinement area, suggesting that as plaques become denser, movement within them is progressively restricted (Spearman *r* = −0.33, *p* = 0.052). *n* = 41 plaques from 4 mice.

We investigated whether differences in the density or morphology of amyloid plaques influence extracellular diffusion and core penetrability. Recent studies have introduced methods to quantify plaque phenotypes using Thioflavin‐S (ThS) staining combined with circularity analysis. This approach yields quantitative and qualitative indicators of plaque size diversity, offering robust and consistent results in both human tissue and genetic mouse models (see the Experimental Section for details).^[^ ^15^, ^24^ ^]^ We applied the same analysis to the APP/PS1 plaques (**Figure** **4**a), assigning a circularity value to each plaque. Since ThS binding may interfere with QD penetration and signal, we developed a post‐hoc labeling protocol: amyloid cores were first localized (based on their autofluorescence under UV light excitation), their coordinates recorded, and QDs tracked. After several recordings, the slice was incubated with 0.001% ThS in aCSF within the recording chamber. Finally, z‐stacks of the previously studied (QD tracking) plaques were captured.

**Figure 5:**
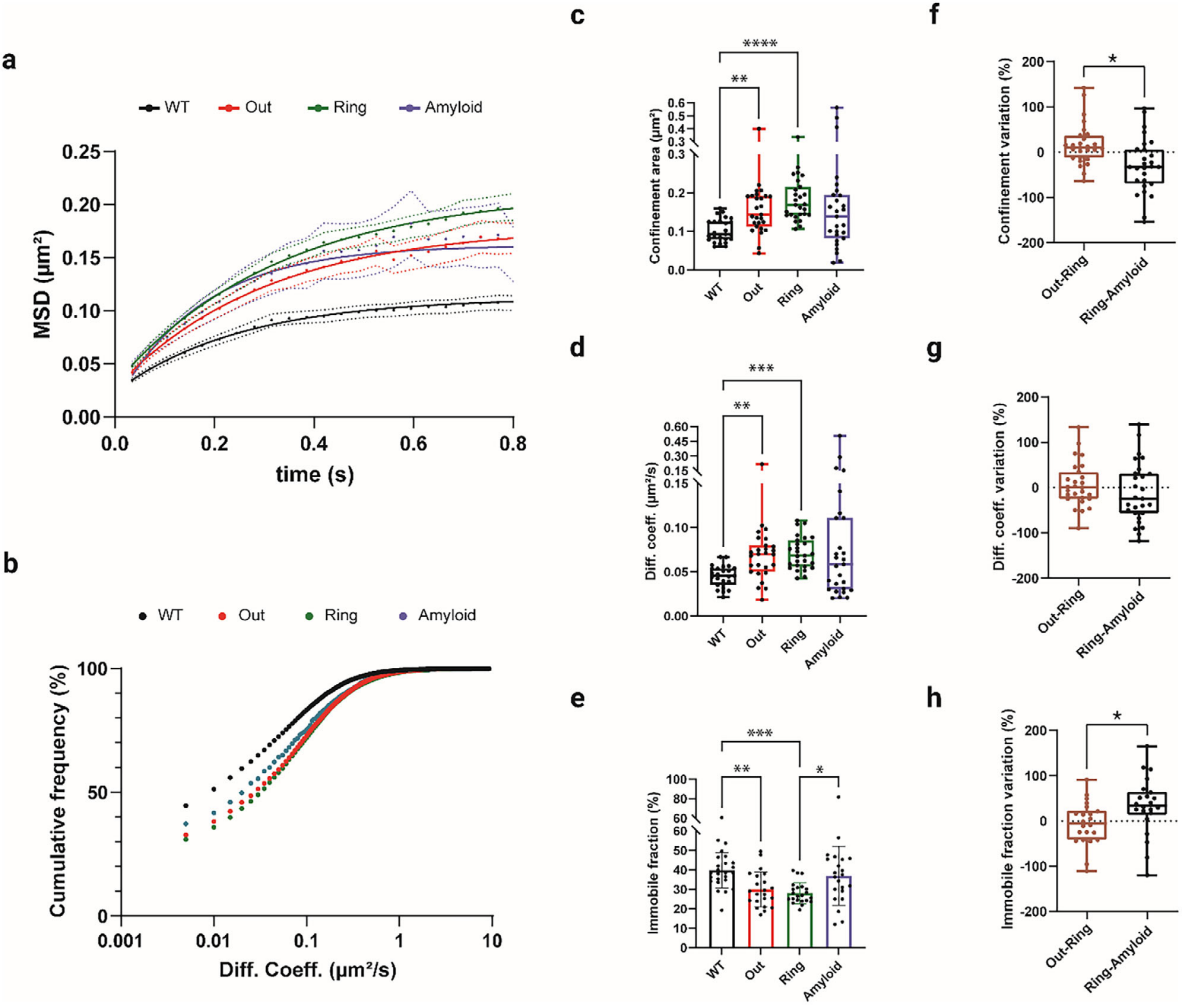
The rheological properties of the ECS differ between wild‐type tissue, around and within plaques. a) The mean square displacement (MSD) curve suggests limited particle diffusion within the ECS. The confined area was estimated to indicate particle exploration (Fitted curve plateau: WT = 0.1123, Out = 0.1792, Ring = 0.2127, Amyloid=0.1617). b) The cumulative frequency distribution of all particle velocities (log scale) was left‐shifted (slower mobility) in the WT (black) compared to the three compartments (colored curves) around and within plaques. c) Comparison of confinement as a proxy of explored ECS area at MSD curve plateau (0.6–0.8 s). In the surroundings of amyloid deposits, but not within it, a significant increase in confinement was observed compared to WT cortices (WT: Median = 0.093, IQR = 0.079–0.125, Out: Median = 0.143, IQR = 0.113–0.192, Ring: Median = 0.169, IQR = 0.143–0.215, Amyloid: Median = 0.139, IQR = 0.082–0.194; Kruskal–Wallis test (Dunn's multiple comparisons correction) *p*‐values: WT vs Out = 0.0054, WT vs Ring <0.0001, WT vs Amyloid = 0.069, Out vs Ring = 0.58, Out vs Amyloid >0.99, Ring vs Amyloid = 0.08). d) Likewise, the medians of diffusion coefficient indicate higher particle velocities in the AD model brain than in WT (WT: Median = 0.045, IQR = 0.035–0.053, Out: Median = 0.07, IQR = 0.050–0.08, Ring: Median = 0.068, IQR = 0.055–0.085, Amyloid: Median = 0.058, IQR = 0.03–0.11, Kruskal–Wallis test (Dunn's multiple comparisons tests), *p*‐values: WT vs Out = 0.0021, WT vs Ring = 0.0002, WT vs Amyloid = 0.075, Out vs Ring >0.99, Out vs Amyloid >0.99, Ring vs Amyloid = 0.54). *N*_WT_ = 25 FOV, from 4 mice; *N*_APP/PS1_ = 27 plaques, from 5 mice. e) Quantification of the immobile fraction of particles highlights an overall smaller fraction in the cortical neuropil (Out) of AD mice compared to controls, with an even greater difference in the cell‐dense ring (WT: Median = 38.4, IQR = 34.5–45, Out: Median = 28.2, IQR = 23.1–37.2, Ring: Median = 26.7, IQR = 24.2–30.9, Amyloid: Median = 36.9, IQR = 28.1–45.6; Kruskal–Wallis test (Dunn's multiple comparisons) *p*‐values: WT vs Out = 0.0043, WT vs Ring = 0.0003, WT vs Amyloid >0.99, Out vs Ring >0.99, Out vs Amyloid >0.23, Ring vs Amyloid = 0.036). *N*_WT_ = 25 FOV, from 4 mice; *N*_APP/PS1_ = 22 plaques, from 5 mice. Error bars represent mean ± SD. f) Paired analysis demonstrates that relative differences in explored area values during the transition from out to ring distributed around zero, in contrast with the transition from cell ring to amyloid plaques, where they assumed negative value (Out–Ring: Median = 10, IQR = −13–37, Ring–Amyloid: Median = −33, IQR = −70–6; Wilcoxon pairs signed rank test, *p*‐values = 0.01). g) Paired analysis of diffusion coefficient variation in the transition between the two adjacent areas showed a comparable trend, indicating that median diffusion also decreases from ring to amyloid. Such a decrease is not statistically significant from that between out and ring (Out–Ring: Median = 0, IQR = −25–34, Ring–Amyloid: Median = −25, IQR = −57–31; Paired *t*‐test, *p*‐values = 0.27). h) Finally, the extent of trapping within the plaques was evaluated through the relative variation of the immobile fraction in successive compartments. Again, the passage from ring to amyloid resulted in a greater variation (increase in this case) in the number of immobile particles (Out–Ring: Median = −5.5, IQR = −42–23.5, Ring–Amyloid: Median = 34, IQR = 13.7–64.7; Paired *t*‐test, *p*‐values = 0.044). *N*_APP/PS1_ = 27 plaques, from 5 mice. All box‐and‐whisker plots display the full range (minimum to maximum).

To investigate the relationships among the experimental variables, we performed principal component analysis (PCA), which revealed a clear association between circularity and impenetrability of the amyloid (QD ratio #locs/area, as defined in the Experimental Section). However, all diffusional parameters were oriented perpendicularly to circularity, suggesting a lack of linear correlation (Figure 4b). We correlated and plotted circularity and QD ratio #locs/area (Figure 4c). Circularity significantly correlates with QD ratio, showing that more circular (denser) plaques exhibit reduced QD penetration, consistent with limited antibody accessibility reported by others.^[^ ^15^ ^]^ Despite the lack of a strong linear relationship in the PCA, we found an almost significant negative correlation (Figure 4d) between circularity and confinement area (see the Experimental Section and **Figure** **5**a for definition), indicating there is a trend that as plaques densify, both penetration and movement within them become increasingly restricted.

Finally, a strong negative correlation exists between highly circular plaques and area, supporting the hypothesis proposed by Condello et al. that denser amyloid plaques represent an advanced and more compact stage of fibrillar plaques.^[^ ^23^ ^]^ However, plaque size alone is not a reliable predictor of the penetrability of the amyloid core (Figure S8, Supporting Information).

### 2.4 Rheological ECS Properties within and around Amyloid Plaques

Beyond penetrability, extracellular rheological parameters are of great relevance when studying the ECS. Mean square displacement (MSD) analysis provides rheological information, i.e., diffusionality and dimensions, and ECS nanoscale properties from SPT experiments in biological media.^[^ ^25^ ^]^ We plotted MSD curves and diffusion coefficient distributions of super‐resolved QD trajectories within and around amyloid plaques over time. On the one hand, diverse curve plateaux were observable at a lag time of 0.4 s, indicating that particle displacement within the ECS was limited to a typical explored area (Figure 5a). On the other hand, the distribution of diffusion coefficients in the ECS exhibited marked differences between experimental conditions (Figure 5b). To quantify these differences statistically, we extracted (from MSD plateaux between 0.6 and 0.8 s) the average confinement area to estimate the space the diffusing particles explored within a given time interval. From both MSD curves and the distribution of confinement values, a dramatic increase in the confinement area was observed in the cortex of APP/PS1 mice compared to age‐matched WT controls (Figure 5c). By contrast, the distribution of confinement values within the amyloid core was not significantly different from WT tissue. Still, the distribution was much wider, likely reflecting other physicochemical differences and amyloid phenotype influence, with a prevalence of extreme values, indicating a significant change in particle mobility within fibrillary aggregates (Figure 5c). QD dynamics were further addressed by the analysis of diffusion coefficient distribution (see the Experimental Section). We set a threshold value (0.005 µm^2^ s^−1^) to study separately the mobile and immobile fractions of particles within the ECS at each level.^[^ ^26^ ^]^ The median diffusion coefficient of the mobile fraction was higher outside the amyloid plaque and in the cell ring in the AD model than in age‐matched WT cortical tissue (Figure 5d). Although not statistically significant, the distribution of the diffusion coefficient was wider for the amyloid region, suggesting alterations in the ECS. Interestingly, both the cortical neuropil (Out) and cell ring (Ring) of APP/PS1 mice exhibited a significantly smaller fraction of immobile particles compared to WT tissue (Figure 5e). Among the few particles that had penetrated the amyloid core, a sizable percentage was immobile, more so than in the surrounding ring (Figure 5e).

We deepened our confinement analysis by pairing values for the three regions (Out, Ring, and Amyloid) obtained from the same field of view. Again, we focused on the two interfaces (Out–Ring and Ring–Amyloid) for which we calculated distributions of their differences expressed as relative change (%) with respect to the average value of each of the two adjacent regions (see the Experimental Section) for all parameters as above. Interestingly, the explored area, diffusion velocities, and immobile fraction displayed substantial variations between the two interfaces. For these three parameters, the percentage of relative change from the outer zone to the ring (Out–Ring) is distributed around zero, which means no variation among neighboring regions. By contrast, at the ring‐to‐amyloid interface (Ring–Amyloid), we observed marked differences between the compartments. Confinement area and diffusion coefficient variations settled around a negative value (i.e., rheological parameters became lower inside the plaques), while the immobile fraction variations were elevated inside the plaques (Figure 5f–h).

Altogether, our approach unveiled substantial nanoscale alterations in the rheological properties of APP/PS1 mouse cortex compared to age‐matched WT. These pathological changes resulted in the unexpected diffusion behavior of particles proximal to Aβ plaques, where mobility was facilitated even within the cell‐dense ring. In parallel, we found a reduction in the availability of nanoparticles within the plaque. Finally, this reduction was more accentuated in the transition from ring to amyloid.

Finally, there is a current controversy regarding the in vivo existence/extent of active flow in the brain parenchyma.^[^ ^27^, ^28^ ^]^ To test if our ex vivo model resembles in vivo diffusion, at least regarding MSD shape, we performed in vivo tracking of QDs using 2‐photon microscopy in WT mice. After the topical application of QD‐containing solution on the mouse cortex, followed by rinsing, we recorded in vivo trajectories in depths ranging from 20 to 40 µm. The analysis shows that most particle diffusion exhibits a restricted/subdiffusive profile that resembles our observations in ex vivo settings with a clear MSD plateau after 1.2 s, supporting the validity of ex vivo experimentation (Figure S9 and Videos S8,S9, Supporting Information).

### 2.5 Cortical Amyloid Plaques Exhibit a Degraded Extracellular Matrix

**Figure 6:**
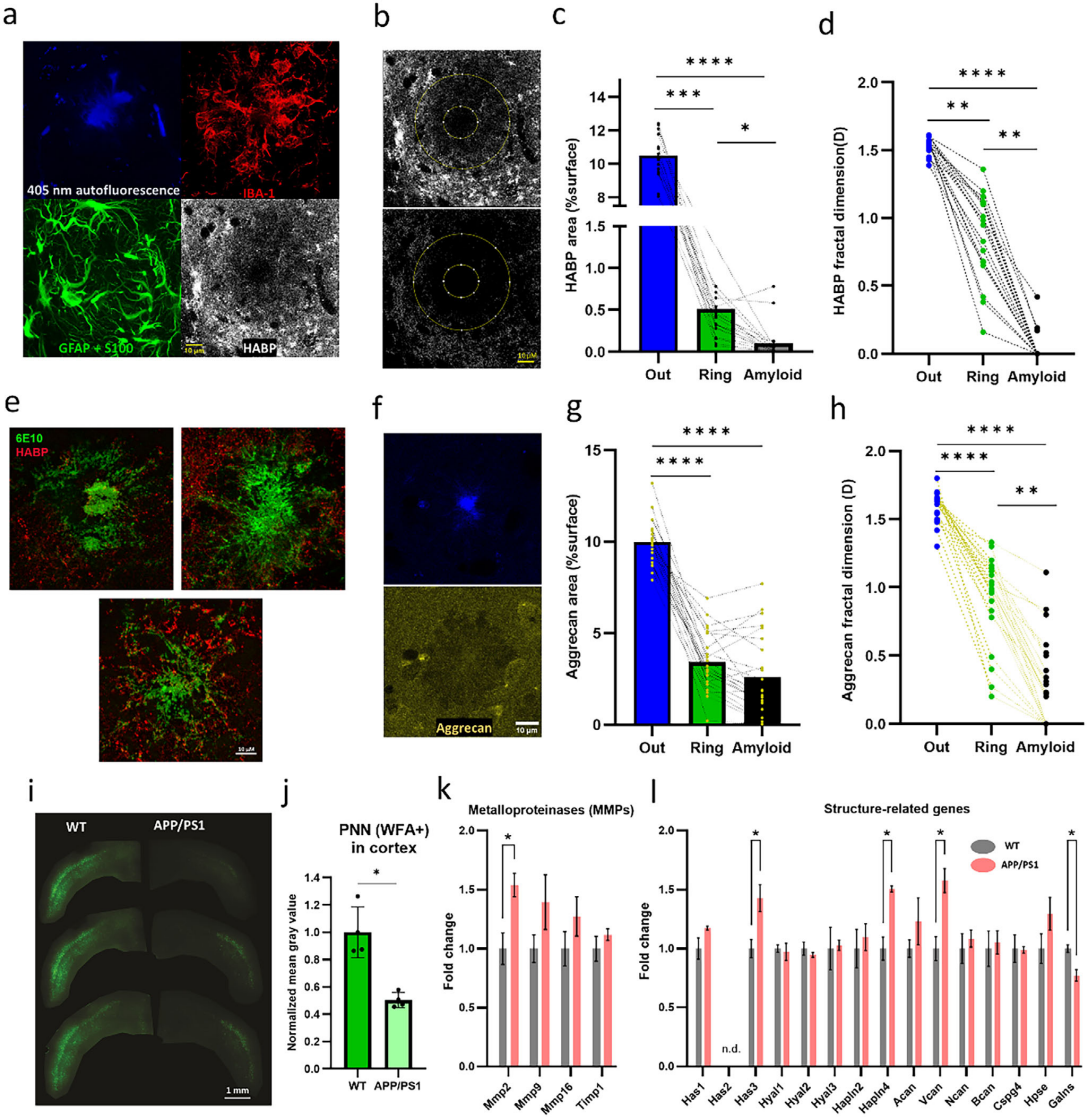
The APP/PS1 mouse presents a highly modified extracellular matrix. a) Amyloid plaques are surrounded by activated astrocytes (GFAP) and microglia (IBA1). Importantly, they also present a highly disrupted Hyaluronic Acid (labeled by HABP) matrix scaffold. b) Matrix thresholding and skeletonization. Matrix is practically nonexistent in the amyloid ROI and is severely degraded in the ring ROI. For segmentation, we applied the same ROIs as in Figure 3. c) Quantification by percentage of HA signal per unit area (%) (Ext. Median = 10.50, IQR = 9.52–11.90; Ring Median = 0.371, IQR = 0.253–0.566; Amy. Median = 0.0175, IQR = 0.0027–0.0750, Kruskal–Wallis test *p* < 0.0001 (Dunn's multiple comparisons correction) *p*‐values: Out vs Ring = 0.0004, Out vs Amyloid <0.0001, Ring vs Amyloid = 0.021). d) Fractal analysis (Ext. Median = 1.56, IQR = 1.52–1.58; Ring Median = 0.96, IQR = 0.66–1.11; Amy. Median = 0, IQR = 0, Kruskal–Wallis test *p* < 0.0001 (Dunn's multiple comparisons correction) *p*‐values: Out vs Ring = 0.0013, Out vs Amyloid <0.0001, Ring vs Amyloid = 0.0022). *N* =18 plaques, from 4 mice. e) Expansion microscopy of amyloid‐β‐immunolabeled plaques (6E10 antibody) and hyaluronic acid (HABP) permits the visualization of the voids and ECM state inside the plaques. f) Aggrecan was labeled and analyzed the same way as HA. g) Quantification by percentage of aggrecan signal per unit area (%) (Ext. Median = 9.90, IQR = 9.40–10.60; Ring Median = 3.10, IQR = 2.36–4.8; Amy. Median = 1.73, IQR = 1.21–4.55; Kruskal–Wallis test *p* < 0.0001 (Dunn's multiple comparisons correction) *p*‐values: Out vs Ring = <0.0001, Out vs Amyloid <0.0001, Ring vs Amyloid = 0.57). h) Fractal analysis (Ext. Median = 1.64, IQR = 1.54–1.66; Ring Median = 0.99, IQR = 0.85–1.12; Amy. Median = 0.215, IQR = 0–0.473, Kruskal–Wallis test *p* < 0.0001 (Dunn's multiple comparisons correction) *p*‐values: Out vs Ring = <0.0001, Out vs Amyloid <0.0001, Ring vs Amyloid = 0.031). i) PNNs were labeled with WFA and quantified in the sensorimotor cortex. j) There is a significant decrease in WFA intensity in APP/PS1 mice (Mann–Whitney test *p*‐value = 0.029, *n* = 4 per group). Each data point represents the average gray value per mouse, calculated from three anterior–posterior cortical slices corresponding to the sensory‐motor region. k,l) mRNA expression analysis of ECM‐related genes in cortex homogenates of WT and APP/PS1 mice. APP/PS1 mice present an altered expression pattern of several key regulator components of the ECM. (j) Metalloproteinases (MMPs) expression levels show an increase in the APP/PS1 mouse (MMP2 *p*‐value = 0.032). (k) Matrix structural‐related genes present altered expression patterns, with upregulated (*Has3 p*‐value = 0.033, *Hapln4 p*‐value = 0.015, *Vcan p*‐value = 0.019) and downregulated expression (*Galns p*‐value = 0.022). Two‐tailed Student's *t*‐test, *n* = 3 mice. Error bars represent mean ± SD. The source data are provided as Supporting Information.

Despite the highly crowded environment in the plaque ring, ECS rheological parameters (Figure 5c,d) are consistent with a facilitated diffusion microenvironment. Such a counterintuitive observation led us to question the integrity of the ECM, a major diffusional regulator of the ECS.^[^ ^29^, ^30^ ^]^ ECM alterations have emerged as a common phenomenon in several neurological conditions^[^ ^31^ ^]^ and amyloid animal models,^[^ ^32^ ^]^ being directly related to the observed increase in ECS diffusion.^[^ ^8^ ^]^ We first studied immunolabeled glial cells (IBA‐1 and GFAP+S100) and hyaluronic acid (labeled by hyaluronic acid binding protein: HABP) around the autofluorescent amyloid core. While the ring is primarily composed of astrocytes and microglial cells,^[^ ^33^ ^]^ we report that amyloid plaques present a highly degraded ECM, with a decreasing HABP signal from the outer ring to the core of the plaque (**Figure** **6**a). For consistency, we used the ROI system defined in previous figures (Figure 6b). ECM changes were quantified by assessing the surface occupied by HABP (Figure 6c). To rule out a decrease in total signal due to a lower ECS volume fraction, we also quantify the fractal dimension^[^ ^34^ ^]^ of the HABP thresholded signal, to estimate matrix interconnectivity and organization (Figure 6d), confirming a significant decrease. The dramatic decrease in hyaluronic acid content in the ring and amyloid supports the observation of an increased QD local diffusion (with the immobile fraction decreasing in the ring) despite the apparent crowded cellular environment. Conceivably, it provides an explanation for why extreme diffusional values are found in the amyloid, i.e., QDs penetrating the amyloid meshwork of proteins/organelles would either stick (immobile) or diffuse (highly mobile) in an amyloid devoid of ECM.

We next used expansion microscopy (Figure 6e) of amyloid‐β‐immunolabeled plaques to visualize the voids and ECM state (labeled again by HABP) inside the plaques, a complementary approach to the earlier work on the tissue‐level impact of amyloid fibers in the 5×FAD mouse model of AD.^[^ ^35^ ^]^ Amyloid appeared susceptible to an expansion, resulting from the insertion of the sodium acrylate between amyloid fibers, i.e., in the ECS, likely filled by the dye in the TUSHI and nanoparticles in the QD experiments, respectively. In agreement with the classic description of diffuse and dense plaques discussed before,^[^ ^36^ ^]^ we observed that protein aggregates present a high degree of structural heterogeneity in ECS and ECM between the fibers (Figure 6e), consistent with the variability in the nanoscopic single‐particle tracking data. To expand our ECM analysis, we imaged the proteoglycan aggrecan, one of the main components of the ECM.^[^ ^31^ ^]^ Following the same protocol and analysis for HABP, we also found a decrease in aggrecan signal, albeit more modest, pointing out that matrix disruptions also affect the proteoglycan component of the matrix (Figure 6g,h).

Finally, we aimed to assess whether matrix disruption can be found beyond plaques. Crapser et al. (2020) reported that PNNs are extensively lost in AD patients and the 5×FAD mouse model in proportion to plaque burden.^[^ ^37^ ^]^ Following a similar approach, we labeled PNN with Wisteria floribunda lectin (WFA) and observed a significant decrease in WFA‐binding PNN in the APP/PS1 sensory‐motor cortex (Figure 6i,j), pointing toward a structural disruption or changes in the “sulfation code” in these key structures.^[^ ^38^ ^]^ To further understand matrix alterations in APP/PS1 mice beyond immunostaining, we examined mRNA expression of genes associated with matrix degradation, structure, and metabolism in cortex homogenates of WT and APP/PS1 mice. We studied the expression of the matrix metalloproteinase family (MMPs) (Figure 6k), given its crucial role in matrix regulation and degradation in health and disease.^[^ ^31^, ^39^ ^]^ Several groups have reported increased endopeptidase levels in amyloid mouse models^[^ ^40^ ^]^ and human AD,^[^ ^39^ ^]^ suggesting their involvement in clearing Aβ. Relevant to this work, MMPs degrade and regulate the turnover and remodeling of ECM proteins,^[^ ^41^ ^]^ making them prime candidates for causing disease.^[^ ^31^ ^]^ We found a significant increase in MMP‐2 mRNA levels and a general increase in the other mRNA coding for proteins of the family. The rise in tissue inhibitor of metalloproteinases 1 might reflect a counterbalancing attempt to dampen the consequences of MMP upregulation. It suggests that the overexpression of MMPs could contribute to the reported degradation of the ECM, conceivably through microglial activation.^[^ ^42^ ^]^ Next, we focused on the expression of matrix structural components (Figure 6l). Hyaluronan synthase 3, hyaluronan/proteoglycan link protein 4, and versican showed increased mRNA levels in APP/PS1 mice and chondroitinase mRNA level is downregulated, probably in a compensatory attempt to regulate or repair digested components. In conclusion, we show that amyloid pathology is also reflected in a highly altered expression pattern of several key matrix components/regulators.

### 2.6 Rheological ECS Properties in the Amyloid‐Pathology‐Bearing Cortex

The collected results provide a comprehensive picture of molecular diffusion in the ECS in and around amyloid plaques. Remarkably, we found that amyloid brain rheology around plaques exhibits significant differences compared to WT (Figure 5). Besides, we make the point that ECM alterations are widespread in amyloidosis. This prompted us to investigate further whether an amyloid brain undergoes global alterations in ECS rheological properties, especially an increased diffusion, using a reporter agnostic of the proximity of amyloid plaques.

**Figure 7:**
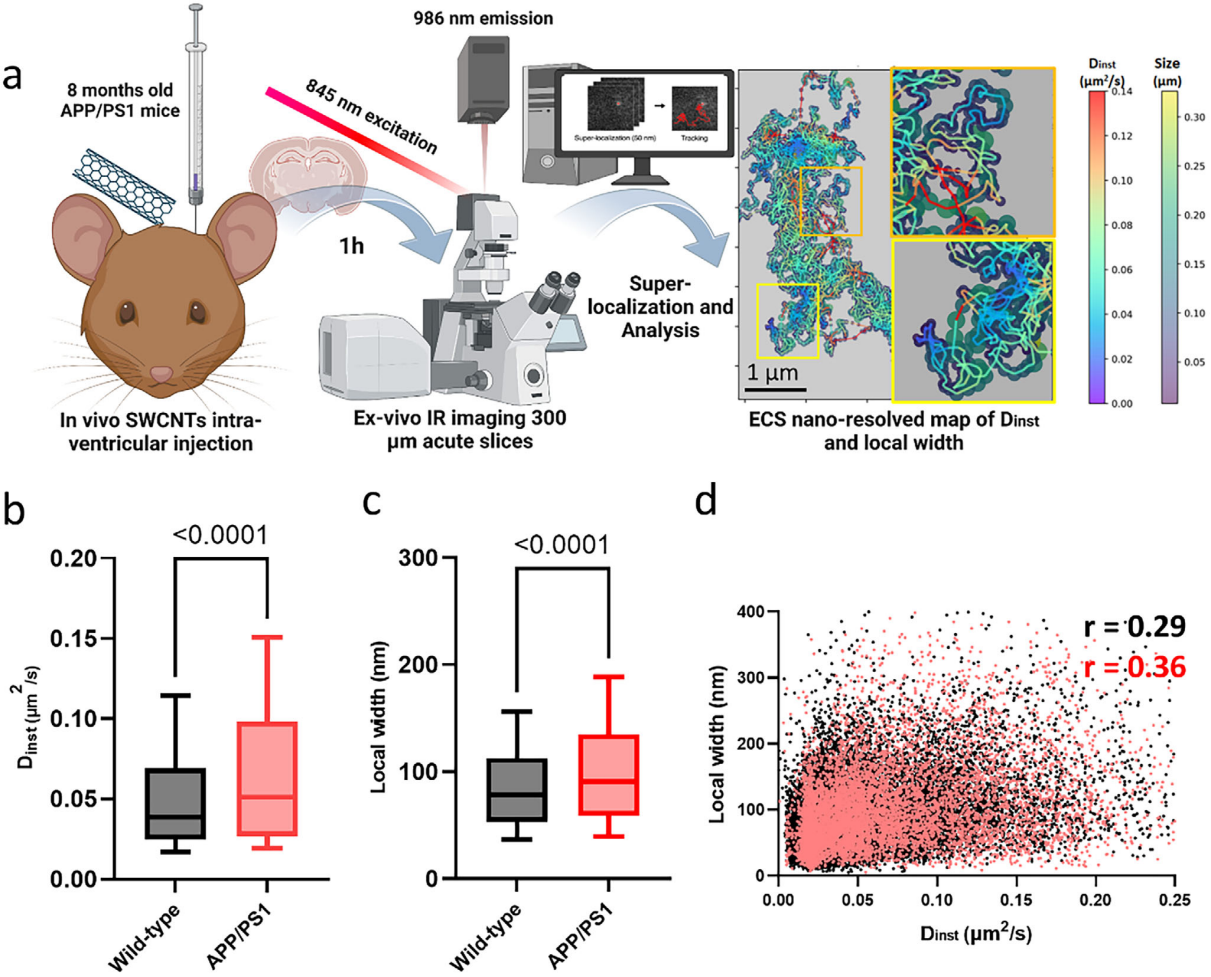
SWCNT tracking reveals a diverse nanoscale ECS and increased local diffusion and ECS width in amyloid pathology. a) SWCNTs are in vivo intraventricularly injected in 8 months old mice and allowed to diffuse into the brain for 1 h. Acute brain slices were prepared in ice‐cold NMDG‐based solution and imaged in aCSF at 37°C under carbogen bubbling. A laser emitting at 845 nm was used to excite (6,5) SWCNTs at their phonon sideband, and their emission peaked at 986 nm. They were filtered using a long‐pass filter before being imaged and recorded with a water‐cooled SWIR InGaAs camera. Individual SWCNTs were superlocalized with a 2D Gaussian fit, and coordinates were linked to reconstruct individual trajectories. Each SWCNT trajectory provides a nanometric‐resolved map of local instantaneous diffusion coefficients (µm^2^ s^−1^) and local widths (nm) (see Figure S12 in the Supporting Information for further details). ECS *D*_inst_ and local size maps reveal a heterogeneous ECS in the brain, with properties that change significantly within a few nanometers. Scale bar = 1 µm. b) Distributions of local *D*_inst_ values increase in diffusion values in the APP/PS1 model (*D*_inst_ (µm^2^ s^−1^) WT Median = 0.038, IQR = 0.025–0.069, APPPS1 Median = 0.051, IQR = 0.027–0.98, Kolmogorov–Smirnov test *p*‐value < 0.0001). c) Distributions of local ECS widths (nm). We show an increase in local width values in the APP/PS1 model (local width (nm) WT Median = 78, IQR = 53–113, APPPS1 Median = 90, IQR = 59–135, Kolmogorov–Smirnov test *p*‐value < 0.0001). Both box‐and‐whisker plots show the distribution from the 10th to the 90th percentile. d) There is a positive but weak spatial correlation between size and *D*_inst_, increasing in pathology (*p* < 0.0001 for both groups, Pearson's *r* = 0.29 WT, 0.37 APP/PS1). *N*_WT_ = 4 mice; *N*_APP/PS1_ = 4 mice. Created in BioRender. Bezard, E. (2023) BioRender.com/i55x918.

Single‐walled carbon nanotubes (SWCNTs) represent a complementary SPT technique using slowly moving near‐infrared reporters, i.e., working in the “transparent” spectral window of biological tissue.^[^ ^43^, ^44^ ^]^ Besides local diffusion coefficients, our analysis based on local restricted theory^[^ ^45^ ^]^ can provide access to the spatial dimensions of the ECS, similar to cryofixed electron microscopy^[^ ^8^ ^]^ and super‐resolution shadow imaging (SUSHI).^[^ ^46^ ^]^ We recently used SWCNTs as super‐resolution imaging probes to report on the local nanoscale organization and diffusivity of the ECS in young^[^ ^45^ ^]^ and adult^[^ ^43^ ^]^ living brain tissue. We also demonstrated that several mouse models of Parkinson's disease were characterized by increased nanoscale diffusion.^[^ ^8^, ^47^ ^]^ Applying this SPT technique to our APP/PS1 mice, we now unravel a heterogeneous cortical ECS, where local diffusion properties change over distances of just a few micrometers and are effectively shaped by brain ECS geometry and composition (**Figure** **7**a). Direct comparison between age‐matched WT and APP/PS1 mice showed an overall increase in local diffusion^[^ ^43^ ^]^ (instantaneous diffusion coefficient (*D*_inst_), Figure 7b). Concomitant with the alterations in local diffusion, we also report a widening of the ECS, i.e., an increase in local channel width based on the analysis of local nanotube trajectories^[^ ^43^ ^]^ (Figure 7c). In agreement with restricted diffusion theory,^[^ ^48^ ^]^ local diffusion and channel width present a weak positive correlation (Figure 7d), suggesting particle diffusion scales with ECS width. However, the modest correlation coefficients indicate that diffusion is a multifactorial parameter, which depends not only on size and geometry. The correlation increases in the amyloid model, pointing to other factors than wider ECS spaces behind the observed increase in diffusion. It raises the question of how a dysregulated ECM affects diffusion in different brain regions. The nuanced relationship between diffusion and channel width underscores the complexity of their interplay. Finally, the SWCNT and QD experiments provide converging evidence that amyloid pathology leads to a more diffusive ECS in the cortical neuropil of APP/PS1 mice compared to WT controls (Figure S10, Supporting Information).

## 3 Discussion

The study employs three advanced imaging techniques, including TUSHI and QD/SWCNT single‐particle tracking, to investigate the morphology and diffusionability, i.e., the rheological properties, of the ECS in and around cortical amyloid plaques, formed by an amyloid core surrounded by a dense ring of cellular components in a transgenic mouse model of AD. The QD‐SPT investigation revealed an increased permeability of the ECS within and around these plaques, but low amyloid core penetrability, depending on amyloid structure, not size. We demonstrate how cortical ECS is remodeled by amyloid pathology and how amyloid plaques display exploitable features for future therapeutic development. These rheological alterations are likely key to understanding the disease. They may contribute to the spreading of the pathology and impact the delivery of therapeutics, as both phenomena are highly dependent on the anatomical fine structure of the tissue and its physicochemical properties.^[^ ^48^ ^]^

### 3.1 Extracellular Space in Amyloid Plaques

Despite its pivotal role in essential processes like synaptic and extrasynaptic communications, extracellular ionic homeostasis, drug delivery, and metabolic waste clearance,^[^ ^29^, ^30^ ^]^ the properties of the ECS have been predominantly overlooked in the scientific literature, partly because of the lack of suitable techniques to address this challenge. Few methods have been developed to measure extracellular diffusion, including diffusion‐weighted magnetic resonance imaging, electrochemical methods, and optical imaging.^[^ ^6^, ^49^, ^50^ ^]^ New optical imaging approaches have since been developed (reviewed in refs.[51, 52]). Among them, SUSHI was designed to address the microanatomical structure of the ECS in living brain tissue with nanoscale spatial resolution.^[^ ^29^ ^]^ Our TUSHI methodology, which is a diffraction‐limited variant of it, enabled the visualization of amyloid plaques deep inside brain tissue. This achievement holds significant importance in the field as it offers a noninvasive means to explore and comprehend the distribution and dynamics of amyloid plaques in their native environment, facilitating the analysis of the intricate interplay of amyloid plaques with the surrounding tissue. Using TUSHI, we observed that small dyes can penetrate the very center of the amyloid plaque. While we could not resolve the internal plaque structure with TUSHI nor obtain ECS volume fractions, the images show that the amyloid plaques are not an impenetrable agglomeration of proteins and organelles but rather a porous‐like mesh formed by amyloid fibrils, sugars, dystrophic neurites, and cellular components that can be penetrated and filled by small molecules, hence the apparent “void” in the diffraction‐limited images (Figure 1; Videos S1–S5, Supporting Information). In addition, it strikingly highlighted the known large and dense ring of cells and neurites that surround the amyloid core like a mantle, increasing by a factor of 25 the effective volume occupied by the plaques. Notably, it also allowed us to define the ROI dimensions required for further exploration with single‐particle tracking.

Proteomic studies that report up to thousands of proteins illustrate the complex composition and crowdedness of amyloid plaques.^[^ ^53^, ^54^ ^]^ Still, although protein, lipidic, and organelle content have received much attention,^[^ ^14^ ^]^ our understanding of how small and larger molecules diffuse in and around amyloid plaques is rudimentary despite its relevance for therapeutics, notably the amyloid‐targeting immunotherapies.^[^ ^55^ ^]^ It is striking that the most advanced quantitative system pharmacology models that describe the nonlinear progression of amyloid ß pathology and the pharmacological actions of the amyloid‐ß‐targeting antibodies do not consider the penetrability of amyloid plaques or their capacity to accumulate exogenous substances.^[^ ^56^ ^]^ While the movement of small molecules and ions is relatively easy to model, understanding how larger objects diffuse within the ECS and interact with ECM components is more challenging due to the multifactorial dependency of extracellular diffusion.^[^ ^29^ ^]^ The single particle tracking methods utilize fluorescent probes diffusing in the ECS, allowing for real‐time monitoring of their trajectories and speeds and for assessing the penetrability of plaques and overall ECS rheology.

### 3.2 Extracellular Probes for SPT: QDs versus SWCNTs

QDs and SWCNTs provide complementary information owing to their specific optical properties and physical dimensions. On the one hand, spherical QDs penetrate the acute brain slice upon incubation. ECS rheology (diffusionality and dimensions) can thus be measured in live tissue with high throughput (many moving particles per field of view). On the other hand, SWCNTs allow thorough exploration of local geometry based on their elongated shape (much thinner but much longer than QDs) and their much better photostability than QDs. More precisely, we took advantage of the fact that the transverse local ECS dimensions laterally constrain the diffusion of the SWCNTs and, therefore, that these dimensions can be locally measured by analyzing the local excursion of the SWCNT.^[^ ^45^ ^]^ In other words, SWCNTs can give access to both local ECS dimensions and local diffusivity. However, SWCNTs do not penetrate the tissue as well as QDs do. The in vivo ICV approach, although elegant, provides a much lower throughput than QDs. This means that, practically speaking, it is complicated to find SWCNTs inside a plaque and even harder to find several in the same field of view to compare the values. In conclusion, the QD method provides shorter trajectories but much higher throughput, while SWCNTs provide better photostability and give access to local dimensions. Besides, we believe that replicating the finding of an increased diffusion in APP/PS1 mice with different probes (which have distinct dimensions and surface chemistry) greatly strengthens our results (Figure S10, Supporting Information).

### 3.3 Extracellular Diffusion: In Vivo versus Ex Vivo Model Relevance

When examining diffusion in the brain, the nature and extent of different types of material and fluid flow become an important question. The emergence of the glymphatic system has introduced the idea of active flow in the brain.^[^ ^57^ ^]^ This type of flow would not be Brownian or passive. Instead, according to the glymphatic system hypothesis, there is an active process that directs and powers fluid flow along specific pathways. In the absence of blood flow and intracranial pressure, the ex vivo conditions do not replicate fully the diffusion/flow situation found in vivo. There is, however, an ongoing controversy regarding the extent of active flow, especially in the parenchyma, where our experiments take place. Many researchers believe that the hydraulic resistance in the narrow channels of the ECS is too high for any bulk flow to occur, even with significant pressure.^[^ ^27^, ^28^ ^]^ To address this in our model, we performed in vivo experiments using a 2‐photon microscope with enough temporal resolution to track and reconstruct QD trajectories. Our results provide crucial proof of concept for the existence of restricted diffusion in vivo, which seems to be predominant in the parenchyma, supporting the robustness of our ex vivo model. However, we must concede that our in vivo recordings are far from optimal for super‐resolution SPT analysis. A relatively big pixel size and lower frequency restrict the accuracy of measurements. Besides, we are limited to the first layer of the cortex, which is likely not representative of the wider cortex. This means that despite the important proof of concept these measurements represent, for the moment, we still rely on ex vivo measurements for quantification.

Besides, our ex vivo approach offers an additional advantage. By disconnecting the presence of putative flows from ECS remodeling, we characterize constitute intrinsic properties of the system. The nanoscale SPT approach measurements provide a “mapping” of the local ECS (local *D*_inst_ can be considered analogous to local viscosity), which is relevant irrespective of any flow considerations.

### 3.4 Factors Controlling Extracellular Diffusion and Penetrability

It is tempting to dissect the different factors affecting diffusion and plaque penetrability. Although amyloid plaques exhibit a dense ring of cellular components, QD diffusion in this ring is not impaired. It even appears slightly facilitated, possibly reflecting a remodeled ECS after a loss of ECM. While we have not directly tested the relation between matrix disruption and increased diffusion in this work, we validated this hypothesis in previous work dealing with other conditions.^[^ ^8^, ^13^ ^]^ Others have also reported such consequences of HA disruption using our SPT approaches.^[^ ^58^ ^]^ Hyaluronan digestion or disruption robustly correlates with increased nanoparticle diffusion. However, the influence of ECM on local hydraulic properties remains correlative and not causal in our experiments.

An interesting point concerns QD density: despite matrix disruption in the “ring” region of the plaque, we report a similar QD density outside the plaque. Even if rheology is facilitated by a dysregulated matrix, cellular components reduce the available extracellular space and, thus, the likelihood of finding a particle and QD accumulation. This equilibrium between low resistance and low available space likely renders the final QD density in each region.

In the amyloid core, diffusion and access are restricted in a plaque‐dependent manner, likely due to size‐selective barriers: we show amyloid core penetrability is highly dependent on plaque compaction, defined by circularity,^[^ ^15^ ^]^ rather than plaque size, rendering certain plaque phenotypes significantly more accessible to antibodies than others. Several studies have shown microglia mutations and relative populations affect the amount the relative number of each kind of plaque, affecting toxicity.^[^ ^15^, ^59^ ^]^ This suggests that a temporal window for successful immunotherapy against plaques exists before microglial‐mediated plaque compaction might render treatments less effective. After such a stage, immunotherapy should be assisted by microglial‐directed therapy (cf. lixisenatide reports on the suppression of microglial reactivity).^[^ ^60^ ^]^

Beyond plaque accessibility, our data suggest a cortex‐wide reorganization of the ECS with widening spaces and increased diffusion. The widening of brain ECS in AD has been implicated in the dissemination and propagation of misfolded/aggregated Aβ and tau proteins, allowing the misfolded proteins themselves, or extracellular vesicles containing them, to propagate from regions of initial pathology to other brain areas later affected during disease progression.^[^ ^61^, ^62^, ^63^ ^]^ It can disrupt physiological synaptic transmission and neuronal communication.^[^ ^64^ ^]^ This disruption, in turn, may contribute to the spread of pathological proteins by altering the microenvironment of neurons and facilitating their vulnerability to protein aggregation.^[^ ^65^ ^]^ The ECS also plays a crucial role in interstitial fluid dynamics, which is essential for the clearance of metabolic waste products, including Aβ and tau. ECS width changes can impact interstitial fluid flow, potentially leading to impaired clearance and the accumulation of pathological proteins, as suggested by others.^[^ ^66^ ^]^ Finally, ECS widening is often associated with neuroinflammatory responses in AD,^[^ ^67^ ^]^ which, in turn, may exacerbate the spread of pathological proteins by promoting cellular damage and dysfunction, creating an environment conducive to the propagation of Aβ and tau pathologies. These hypotheses can now be thoroughly tested using the developed technologies.

### 3.5 The Mouse Model: Relevance and Limitations

Further exploring how plaque composition, maturity, and morphology influence drug penetration could inform personalized treatment strategies tailored to the stage of AD progression. Here, however, we used a transgenic mouse model of AD, the APP/PS1 mouse model. Since 1995, more than 100 transgenic mouse models of AD have been developed expressing high levels of mutant human amyloid precursor protein, showing varying degrees of symptoms, pathophysiological processes, and plaque formation.^[^ ^68^ ^]^ While the Tg2576, APP23, APP/PS1, and 5×FAD lines are popular in the literature, their validity remains debated.^[^ ^69^ ^]^ The pathological changes in APP/PS1 mice show several similarities with AD: the abundant age‐dependent severe neuropathology^[^ ^70^ ^]^ is associated with global brain atrophy,^[^ ^71^ ^]^ decreased glucose metabolism in the hippocampus,^[^ ^72^ ^]^ and complex AD‐like cognitive^[^ ^73^ ^]^ as well as noncognitive behavioral manifestations.^[^ ^74^ ^]^ The model inevitably also presents discrepancies with AD. For example, it is based on overexpression of APP, which does not happen in human AD and Aβ production is limited to specific cell types (a limitation that has led to the third generation of mouse AD models, the KI lines^[^ ^75^ ^]^), while hyperphosphorylated tau remains at low levels, is distributed in a different way than in human AD patients, and does not form neurofilaments.^[^ ^76^ ^]^ The lack of tau pathology is held responsible for the limited neuronal loss and lower amyloidogenic processing of the APP as compared to humans.^[^ ^77^ ^]^ Finally, the proportion of diffuse versus dense‐core plaques in our mice appears to differ from that reported in sporadic human AD, with mice tending to show a lower proportion of fibrillar plaques.^[^ ^78^ ^]^

### 3.6 Study Limitations, Proposed Future Experiments, and Potential Applications

This study underscores the importance of the dynamic nature of AD pathology for developing improved therapeutic strategies. Not only does it offer a screening platform for measuring the penetrability of said experimental therapeutics in amyloid plaques, but it also paves the way for elaborating nanotechnology‐based approaches, such as targeted drug delivery systems or modulators of ECS dynamics, to improve drug penetration and efficacy for advancing AD therapeutics. Notably, functionalized QDs represent a powerful approach to mimic antibodies in size and cell interactions. However, a limitation of the current setup is the simultaneous shadow imaging and SPT data acquisition, preventing the parallel study of the same plaque. Technically, integrating shadow imaging, particularly in combination with STED, alongside SPT, represents a challenge. This approach would allow for concurrent analysis of cellular architecture and dynamic processes, offering deeper insights into the relationship between mechanical and structural features and their impact on biological functions. Specifically, it would be highly valuable to link plaque rheology and penetrability with amyloid aggregation and different amyloid morphologies.

The QD‐SPT technique we propose could answer other biologically meaningful questions. For instance, tumors represent compelling structures for extracellular diffusion measurements using our QD‐SPT protocol. Like amyloid plaques, tumors present a particular tumor microenvironment characterized by altered ECM and diffusion.^[^ ^79^ ^]^ ECM remodeling—marked by increased hyaluronic acid, tenascin‐C, tissue stiffness, and MMP activity—supports tumor cell migration, immune suppression, angiogenesis, and treatment resistance, making it a critical target for therapy.^[^ ^80^ ^]^ Efficient drug delivery remains a significant challenge in tumor nanomedicine, as many systems are trapped in the tumor ECM rather than penetrating deeper.^[^ ^81^ ^]^ Computational and in vitro studies^[^ ^79^ ^]^ show that nanoparticle diffusion decreases with increasing ECM density and stiffness, yet the experimental literature on this topic is limited. Our QD‐based approach offers a promising solution for studying diffusion in such microenvironments. Additionally, live‐tissue dyes^[^ ^82^ ^]^ (e.g., 5‐ALA, fluorescein sodium, ICG) can complement our method for straightforward diffusion measurements in live tissue. In addition, the simplicity of QD surface modification makes them ideal for improving drug delivery. As dyes and reporters used in our imaging techniques are bath‐applied, it is, therefore, possible to explore ex vivo in surgically resected human brain tissues or postmortem samples collected with a reasonable time lag to validate the penetrability of genuine AD plaques or tumors by reporters with dimensions comparable to therapeutics.

In conclusion, our study provides new insights into the factors that influence the penetration of exogenous molecules into Aβ plaques themselves and Aβ‐plaques‐bearing cortex in experimental AD. By elucidating the dynamics of the ECS in and around plaques, the findings advance our understanding of this devastating neurodegenerative disorder.

## 4 Experimental Section

### Animals

Experiments were performed following the European Union directive (2010/63/EU) on protecting animals used for scientific purposes. They were approved by the Ethical Committee of Bordeaux University (CE50, France) and the Ministry of Education and Research under license number APAFIS #32540‐2021072016125086 v11. The study was reported following ARRIVE guidelines (https://arriveguidelines.org).

8 months old female APPswe695/ PS1ΔE9, termed APP/PS1 (Stock number: 005864), obtained from Jackson Laboratory (Bar Harbor, ME, USA), and their WT littermates (C57BL6/J) were used. Plaque size diversity (Figure 4) was investigated at the revision stage using a batch of animals from the same source (4 APP/PS1 animals, female aged 7/10 months). Briefly, the APP/PS1 mice expressed a chimeric mouse/human amyloid precursor protein APPswe (mouse APP695 harboring a human Aβ domain and mutations K595N and M596L linked to a Swedish familial AD) and a human presenilin 1 mutated in familial AD (PS1ΔE9; deletion of exon 9). These bigenic mice were created by coinjection of both transgenes allowing for a cosegregation of the transgenes as a single locus.^[^ ^83^ ^]^ Mice were generated in the animal facility from double‐transgenic APP/PS1 males mated with C57BL/6 J females. Transgenic mice (APP/PS1) and age‐matched nontransgenic littermates (WT) were allowed free access to food and water and maintained in a 12 h dark–light cycle. Mice were genotyped and systematically genotyped after each experiment (Transcriptomics Platform, Neurocentre Magendie, Bordeaux, France). In all experiments, females were used regardless of their estrous cycle.

### In Vivo TUSHI 2‐Photon Microscopy

Animals were injected with buprenorphine (0.1 mg kg^−1^) before the surgery for pain relief. Surgery was done under isoflurane anesthesia. A round craniotomy (≈4 mm in diameter) left the dura mater intact, and was made above the somatosensory cortex. The dye (Alexa Fluor 488, carboxylic acid, Invitrogen, 3–4 µL with a concentration of 50 mm) was injected into the lateral ventricle on the side of the craniotomy at coordinates: M/L‐1.1, A/P‐0.5, D/V‐2.3 at a rate of 500 nL min^−1^ using a motorized syringe pump. The craniotomy was covered with a glass coverslip (#1 thickness, diameter 4 mm) and sealed with glue and dental cement (Superbond C&B). After surgery, mice were anesthetized with ketamine/xylazine (100/10 mg kg^−1^) and placed on a heated blanket under the objective of a custom‐built 2‐photon microscope, based on a commercial upright research microscope (BX51WI, Olympus, Hamburg, Germany) equipped with a 60× silicone oil objective (UPLSAPO, NA 1.3 Olympus) mounted on a *z*‐axial nanopositioner (Pifoc 725.2CD, Physik Instrumente). 2‐photon excitation was achieved using a femtosecond mode‐locked fiber laser (Alcor 920, Spark lasers) delivering <100 fs pulses at a wavelength of 920 nm and a repetition rate of 80 MHz. Laser power was adjusted via a Pockels cell (302 RM, Conoptics) to up to 30 mW of power after the objective depending on imaging depth, and the pixel dwell time was 100 µs. The epifluorescence signal was descanned and detected by an avalanche photodiode (SPCM‐AQRH‐14‐FC, Excelitas). Signal detection and hardware control were performed with the Imspector scanning software (Abberior Instruments) via a data acquisition card (PCIe‐6259, National Instruments).

### In Vivo QD Tracking by 2‐Photon Microscopy

Surgery was performed under isoflurane anesthesia immediately before imaging. Animals were injected with buprenorphine (0.1 mg kg^−1^) before the surgery for pain relief. A round craniotomy (1.2 mm in diameter) was made above the somatosensory cortex to expose the brain's surface. Mice were placed on a heated blanket under the objective, and the head was fixed with a SGM‐4 Head Holder for Mice (Narishige). 100 µL of Qdot 655 (Q11422 MP, Invitrogen) solution (1 mm in aCSF) was placed over the brain surface and left for 5 min to diffuse into the tissue. The surface was then rinsed using physiological serum. Imaging was performed an Olympus BX61WI microscope (Olympus, Tokyo, Japan) equipped with a Nikon Apo LWD 25X water‐immersion objective (NA 1.10). For 2‐photon excitation, a Chameleon Vision 2 laser (Coherent, Santa Clara, CA, USA) was employed. The wavelength was set at 840 nm. Laser power modulation was controlled by a Pockels Cell (Conoptics, Danbury, CT, USA) in conjunction with a half‐wave plate. Imaging was performed using the galvo scanner pathway, controlled by MESc software (Femtonics, Budapest, Hungary). Fluorescence signals were detected using a nondescanned GaAsP photomultiplier tubes (PMTs) equipped with red emission filters for signal collection. Acquisitions were performed between 20 and 40 µm in depth. Videos were cropped based on intensity profiles using Python to eliminate the breathing effect and frames were 3‐frame rolled averaged to improve signal. Analysis was performed with PALMtracer as described below.

### Acute Brain Slice Preparation for Ex Vivo Imaging

Mice were euthanized by cervical dislocation. Brains were swiftly extracted, and coronal sections (300 µm thick) were prepared in a VT1200S vibratome (Leica) in ice‐cold NMDG solution and left to recover in NMDG solution for at least 20 min at room temperature. NMDG in mm: NMDG (93), KCl (2.5), NaH_2_PO_4_·2H_2_0 (1.2), *N*‐(2‐hydroxyethyl)piperazine‐29‐(2‐ethane‐sulfonic acid) (HEPES) (20), glucose (25), NaHCO_3_ (30), MgSO_4_ (10), CaCl_2_ (0.5). Sodium pyruvate (1) and *N*‐acetylcysteine (12) were added just before the experiment. pH was adjusted to 7.3–7.4 using HCl. Measured Osm 290–300. Slices were then transferred to room temperature aCSF (gassed with 95% O_2_, 5% CO_2_) with the following composition (in mm) NaCl (140), KCl (2.5), NaH_2_PO_4_·2H_2_O (1.2), NaHCO_3_ (26), glucose (10), HEPES (10), MgSO_4_ (2), and CaCl_2_ (2). Sodium pyruvate (1) and *N*‐acetylcysteine (12) were added just before the experiment. pH was adjusted to 7.3–7.4 using HCl. The osmolarity was 300–310 mOsm.

### Ex Vivo TUSHI 2‐Photon Microscopy

Acute brain slices were imaged using a commercial 2‐photon microscope (Prairie Technologies). For imaging of Calcein (200 µm, bath applied in aCSF composition: NaCl (126), KCl (3.5), NaHCO_3_ (25), glucose (12), NaH_2_PO_4_•2H_2_0 (1.2), MgCl_2_•6H_2_0 (1.3), and CaCl_2_•2H_2_0 (2). Sodium pyruvate (1) and *N*‐acetylcysteine (12), pH 7.3–7.4. The osmolarity was 300–310 mOsm. The wavelength of the 2‐photon laser (Ti:sapphire, Chameleon Ultra II, Coherent) was tuned to 810 nm. Images were acquired using a 40× water immersion objective with a NA of 1.0 (Plan‐Apochromat, Zeiss). Laser power was between 10 and 25 mW after the objective. The nondescanned fluorescence signal was collected by a PMT detector. Images were acquired with a pixel size of 144 nm over a 295 × 295 µm^2^ field of view (FOV) and pixel dwell times between 15 and 25 µs. Pixel size was selected based on the Nyquist sampling theorem, ensuring adequate spatial resolution. Image acquisition was controlled by the Prairie View software.

### TUSHI Image Processing and Data Analysis

TUSHI images were visualized and analyzed using ImageJ. ROIs were manually selected using the “free‐shape” drawing tool and the surface area of amyloid and crown was calculated (Figure 2a). The average total radius and average radius of amyloid (*r*) were calculated from the surface area (*A*) of amyloid + ring and amyloid, respectively, approximated by a circle from the equation *A* =  π*r* ^2^. The thickness of the ring was calculated as the difference of the total radius and the radius of the amyloid. All data in the text were represented as median and IQR.

### QD Characterization

Because of their proven biocompatibility, commercial photoluminescent Qdot 655 (Q11422 MP, Invitrogen) was used.^[^ ^17^ ^]^ These particles were passivated by a PEG corona, further decorated with F(ab′)2 IgG (H+L, goat anti‐rabbit) fragments to confer antibody‐like diffuse behavior to the particles. Scanning transmission electron microscopy (Talos F200S G2–Thermo Fisher Scientific Inc.) combined with a cationic negative staining protocol was used to image the nanocrystals' dry size and the surrounding organic material (Figure S3, Supporting Information). Briefly, QDs were diluted in HEPES buffer (NaCl (150 mm), HEPES (50 mm); pH 7.5) to a final concentration of 100 nm. A solution of uranyl acetate (UA) in diH_2_O (1.5% w/v) was freshly prepared, protected from light, and filtered with a 0.22 µm cutoff. A 10 µL drop of QD suspension was loaded on carbon grids (1 min, CF150‐Cu‐50–Delta Microscopies), serial washes in diH_2_O (×3) followed, and UA negative staining was performed (1 min). A single wash in diH_2_O was finally done after staining (note: between each step, the liquid was removed through absorption by capillarity with filter paper). QDs on grids were imaged at accelerating voltages of 200 keV and visualized with a 4K × 4K camera (One View–Gatan). The hydrodynamic size of QDs was studied by DLS (Zetasizer µV–Malvern Panalytical). Nanoparticles were diluted in HEPES buffer to a final concentration of 50 nm and 50 µL of suspension was placed in a disposable cuvette. This latter was loaded and equilibrated on the device for 120 s at 20°C. Readings consisted of averaging 8 measurements of 20 s repeated 4 times, count rate and correlation function were monitored to select reliable recordings in the absence of thermal drifts, sample agglomeration, and precipitation, as suggested by the manufacturer. Particle size distribution was fitted with a lognormal function to extract geometric mean and standard deviation.

### QD Incubation in Acute Brain Slices

Acute slices were prepared as described above. After preparation and recovery, slices were then incubated for 40 min at 35°C in 5 mL of QD suspension (200 pm) in carbogenated aCSF (95% O_2_, 5% CO_2_, in mm): NaCl (126), KCl (3.5), NaHCO_3_ (25), glucose (12), NaH_2_PO_4_•2H_2_0 (1.2), MgCl_2_•6H_2_0 (1.3), and CaCl_2_•2H_2_0 (2). Sodium pyruvate (1) and *N*‐acetylcysteine (12), pH 7.3–7.4. Measured mOsm 300–310. Slices were rinsed for 8 min in 5 mL of bubbled QD‐free aCSF before imaging.

### QD Time‐Lapse Confocal Microscopy

Samples were placed in a thermostatic recording chamber (35°C–Life Imaging Services GmbH) mounted on an upright microscope (Eclipse Ni‐E, Nikon) equipped with a confocal spinning disk unit (CSU‐X1, Yokogawa). Amyloid plaques in APP/PS1 mice cortices were identified by their autofluorescence under 405 nm (180 mW, 5%) laser diode excitation (L4Cc, Oxxius), while QDs were excited at 488 nm (200 mW, 50%). Excitation light was separated from the emitted using a quad‐band polychroic mirror (405/488/561–568/635–647, Chroma); emitted light was further passed, respectively, on a 447/60 and 655/15 nm single‐band bandpass filter (BrightLine). During time‐lapse particle tracking, slices were continuously perfused with gassed aCSF at a rate of 1 mL min^−1^ (35°C). Cortical areas were identified under 10× magnification (CFI Plan Fluor, 0.45 N.A.), a 60× water dipping objective (CFI Apo NIR, 1 NA) was instead used to image senile plaques and QDs (>15 µm depth). Particle diffusion was sampled at 28 Hz for 1 min using an EMCCD camera (Evolve 512–Teledyne Photometrics). The imaging system was controlled by an integrated acquisition software (NIS‐Elements AR–Nikon). For plaque size diversity identification, recordings were performed as explained above, but keeping the precise location of each plaque. After several QD recordings, Thioflavin S 0.01% was added for 10 min and then rinsed by aCSF perfusion to label amyloid fibers. A *z*‐projection of maximal fluorescence intensities across 10 optical slices (*Z*‐step = 1 µm) was made through the recordings of QDs. The plaque area was selected using the Wand tracing Tool after “isodata” thresholding. The selection was used to calculate circularity using the Fiji parameter “Shape Descriptor” (circularity = 4π × area/(perimeter) 2). Categorical classification was performed, as described in ref.[15]: filamentous plaques had a circularity score of 0.00–0.14, and compact plaques had circularities greater than 0.28. Plaques with circularity scores between 0.15 and 0.28 were classified as having “intermediate” phenotypes.

### QD Localization and Trajectory Analysis

Low‐resolution image stacks of single emitters were segmented, particle position was superlocalized, and single molecule trajectories were reconnected with PALMtracer, a high‐end software package (MetaMorph–Molecular Devices) for the analysis of single‐molecule dynamics. Briefly, a wavelet segmentation and a Gaussian fitting (6 pixels around maxima) were used for threshold‐based localization. At the same time, a simulated annealing algorithm was applied for trajectory reconstruction through successive images. As a result, the MSD of moving particles as a function of time was obtained. Only trajectories described by more than 10 points were analyzed. The *D*_inst_ was calculated for all trajectories through a linear fit of the first 4 points of the MSD curve. At the curve plateau, between 0.6 and 0.8 s, the mean square displacement average value was used as a proxy of the confinement area experienced by QD. To evaluate the extent of trapping within the plaques, the immobile fraction was obtained by counting the number of particles moving at velocities lower than 0.005 µm^2^ s^−1^ over the total (only FOV where Amyloid ROI had at least one immobile QD was considered for immobile fraction analysis). Relative changes of studied parameters in the three (Out, Ring, and Amyloid) anatomical compartments of the plaques were studied as average percentage variation between adjacent ROI in the same FOV: $\frac{\left(x\;-\;y\right)}{0.5\;\times \;(x+y)}\;\times \;100\;$(*x* = outermost, *y* = innermost).

### QD Localization Accuracy

The localization accuracy for QD tracking was measured with the SPT setup described above (see QD time‐lapse confocal microscopy). About 50 frames were recorded to establish an immobile particle's *x* and *y* location within the field of view. Particles with median velocities lower than 0.005 µm^2^ s^−1^ were considered immobile.^[^ ^84^ ^]^ The standard deviation (SD) of particle position in each frame was measured, and the averages between *x* and *y* SD were plotted. Instantaneous diffusion of immobile particles over time was also plotted to highlight the difference between QD immobilization on a glass coverslip (which defines the SPT setup limit of localization accuracy) and immobile QD detected within the living tissue (which concerned the actual experimental localization). See Figure S6 in the Supporting Information.

### SWCNT Preparation and Characterization

SWCNT solution was prepared following the previously described methods^[^ ^43^ ^]^ with some minor modifications. 1 mg HiPco‐synthesized CNT (batch #189.7) was suspended in 0.5% w/v phospholipid (PL)–PEG (#mPEG‐DSPE‐5000, Nanocs) in 10 mL MQ water. The dispersion was typically homogenized for 15 min at 8000 rpm using a dispersing instrument (IKA T‐10 Basic), and then further dispersed by tip sonication at 20 W (output power) for 10 min under the ice. Nanotube bundles and impurities were precipitated by centrifugation at 10 621 *g* for 60 min at room temperature. 70–80% of the supernatant of PL–PEG suspended SWCNTs was then collected and stored at 4°C. The concentration of the CNT stock solution was estimated by UV (EVOLUTION 220, Thermo Scientific). A range of SWCNT concentrations were used (1–10 mg L^−1^). The length of the SWCNTs was measured by AFM. For this, typically, SWCNT solution was drop‐casted on freshly cleaved mica surfaces. Then, the substrate was gently rinsed with water, followed by overnight air drying (Figure S11, Supporting Information).

### SWCNT Intracerebroventricular Injection

6 µL of SWCNT solution (2–10 mg mL^−1^) was injected into the right lateral ventricle of living mice by stereotactic surgery (coordinates from bregma: −0.5 mm anteroposterior, 1 mm mediolateral, −2.4 dorsoventral) as previously described.^[^ ^43^ ^]^ 30 min before surgery, animals received subcutaneously received buprenorphine (0.1 mg kg^−1^) as an analgesic and lurocaine (5 mg kg^−1^) as a local anesthetic. Surgery was performed under deep isoflurane anesthesia. SWCNT solution was injected with a 30 gauge Hamilton syringe coupled to a microinjection pump (World Precision Instruments) at a 1 µL min^−1^ flow rate. The needle was left in place for 12 min to avoid leakage, then slowly retracted, stopping for another 2 min at −1.2 DV. Between 40 min and 1 h after an injection of the nanotubes, mice were euthanized by cervical dislocation.

### Ex Vivo SWCNT Near‐Infrared Microscopy

Acute slices were prepared as described above. After recovery, images were collected at 35°C in a 3D‐printed chamber with controlled temperature by a feedback system. Prewarmed carbogen‐bubbled aCSF was perfused throughout the chamber by a peristaltic pump. Slices were imaged for no more than 45 min. Imaging of moving SWCNTs in the ECS was performed on a customized upright epifluorescent microscope (Nikon). An 845 nm laser diode (QSI), polarized circularly with a quarter‐wave plate (Thorlabs), was used to excite the (6,5) SWCNTs at a phonon sideband. Emission light was collected through a 900 nm long‐pass filter (Chroma) using a 25×/1.10 NA water‐dipping objective (Nikon). Images were acquired with an InGaAs camera (C‐RED 2–First‐Light Imaging) at 33 ms exposure time. A 1.5× zoom magnification was applied to match the camera pixel size with the microscope point‐spread function width. Before recording, transmission white light was used to check the position in the entire slice and determine the brain region to be imaged. To avoid nonphysiological data acquisition, the first 10 µm of tissue was always discarded to exclude the first cell layer, which was potentially damaged during slicing.

### SWCNT SPT Analysis

The analysis was performed as previously described with minor modifications.^[^ ^43^ ^]^ Briefly, individual SWCNTs were superlocalized with a 2D Gaussian fit, and coordinates were linked to reconstruct individual trajectories. Local diffusion and local ECS dimensions were then extracted as previously described in ref.[45] upon drift correction using nonmobile particles in the field of view. For each trajectory, the instantaneous MSD analysis was used to estimate the instantaneous diffusion coefficient *D*_inst_. MSD values were calculated over a sliding window of 10 frames and linear fits were then applied to the first 3 time points to retrieve values of *D*_inst_. Immobile SWCNTs, characterized by the plateau shape of their global MSD, were excluded from the analysis. To estimate local ECS dimensions, the shape of the local area explored by individual SWCNTs along their trajectory was analyzed using a 6‐point time window.^[^ ^45^ ^]^ This time window was chosen at the maximum confinement (i.e., when the shape of the local area is maximally distorted by local ECS dimension as compared to expected in unconfined environments) as defined by the eccentricity ratio of the ellipse formed by the SWCNT trajectory (Figure S12, Supporting Information).

### SWCNT Localization Accuracy

The precision of the measurements was critically influenced by both the number of photons detected and the level of background noise. Three representative trajectories of diffusing SWCNTs were analyzed and the localization precision was extracted using ThunderSTORM,^[^ ^85^ ^]^ as shown in Figure S13 (Supporting Information).

Assuming a median localization precision of 30 nm, the error on the measured instantaneous diffusion coefficients could be estimated owing to this precision. From the equation of the MSD, it was found that the error on the instantaneous diffusion coefficient could be approximated as

(1) $$\Delta D_{inst}∼\frac{{\left(\Delta x\right)}^{2}}{t}∼\frac{{\left(30.10^{-9}\right)}^{2}}{3*30.10^{-3}}∼0.01\mu m^{2}.s^{-1}$$

where Δ*x* is the localization precision and *t* is the time window where Δ*D*_inst_ is measured.

This error was in the order of ≈15% of the median value of *D*_inst_ found in this study.

### Immunostaining and Fluorescence Imaging

Brains were swiftly extracted and fixed in 4% PFA at 4°C for 24 h and rinsed in phosphate‐buffered saline (PBS). Then, 50 µm thick vibratome coronal sections were collected and kept in PBS at 4°C before immunostaining. For visualization of the hyaluronan matrix, were treated with a streptavidin–biotin blocking kit (Vector Labs) for 20 min and incubated overnight with biotinylated‐HABP from bovine nasal cartilage (Merck Millipore) diluted in blocking solution. Staining was revealed with Streptavidin–Atto647N (Sigma‐Aldrich). Double or triple labeling was achieved by reblocking samples with 4% normal goat serum and overnight incubation with primary antibodies for the following antigens: Iba1 (Rb, 019‐19741, Wako), GFAP (Ms, MAB360, Merck Millipore). Staining was revealed with appropriate secondary antibodies conjugated with Alexa 488 or 594 (Thermo Fisher). Aggrecan was revealed by overnight incubation with Aggrecan Polyclonal Antibody (Rb,13880‐1‐AP, Thermo Fisher). PNNs were labeled with WFA (WFL) conjugated with a green fluorophore (L32481, ThermoFisher) by incubation for 6 h. Sections were mounted on #1.5 coverslips with VECTASHIELD Antifade Mounting Medium and left to dry overnight in darkness. Confocal images were acquired in a Leica TCS SP8 microscope with a 63× Plan Apo CS objective with oil immersion, maintaining image acquisition settings (laser power, AOTF, detection parameters) between sessions. Image stacks: pixel size: 90 nm, *z*‐step 0.5 µm. Images were treated and analyzed with Fiji/ImageJ. Wide‐field images (PNN imaging) were acquired in a BX 63 Olympus microscope with a mercury lamp, using a Dry UPLFLN 10× objective (NA 0.3). Acquisition parameters were kept between acquisitions. Analysis was performed in Image/J Fiji. ROIs were drawn manually. Each value was obtained from 3 slices covering the sensory‐motor cortex of mice.

### Expansion Microscopy

Double labeling was achieved on 50 µm thick vibratome coronal sections (HABP from bovine nasal cartilage, Merck Millipore) and β‐Amyloid (Biolegend) (adapted from ref.[86]). Staining was revealed with appropriate secondary antibodies conjugated with Alexa 488 or 568. Samples were incubated overnight at RT with the succinimidyl ester of 6‐((acryloyl)amino) hexanoic acid (acryloyl‐X, SE; Life Technologies), 1/100 in PBS. Gelation was achieved by incubation for 30 min at 4°C in monomer solution (1× PBS, NaCl (2 m), 8.625% w/w sodium acrylate, 2.5% w/w acrylamide, 0.15 w/w *N*,*N′*‐methylenebisacrylamide) was mixed and ammonium persulfate initiator and tetramethylethylenediamine accelerator were added up to 0.2% w/w each and inhibitor 4‐hydroxy‐2,2,6,6‐tetramethylpiperidin‐1‐oxyl up to 0.01% w/w before using. Slides were placed in a chamber at 37°C for 2 h digestion and expansion: gelled samples were then incubated in proteinase K 8 units mL^−1^ in digestion buffer (Tris (pH8, 50 mm), ethylenediaminetetraacetic acid (1 mm), Triton X‐100 (0.5%), guanidine HCL (0.8 m)) for at least 12 h at RT. Digested gels were next placed in excess volumes of doubly deionized water for 2 h to expand. Images were imaged in water in a Leica TCS SP5 with an objective 25× water immersion objective (NA 0.95), maintaining image acquisition settings (laser power, AOTF, detection parameters) between sessions. Image stacks: pixel size: 110.2 nm, *z*‐step optimized by software. Images were treated with Fiji/ImageJ.

### Quantitative Real‐Time Polymerase Chain Reaction (qPCR)

Total RNA was isolated using the Quick‐RNA FFPE Kit (ZYMO research). RNA was processed and analyzed following the MIQE guidelines.^[^ ^87^ ^]^ cDNA was synthesized from 0.5 µg of total RNA by using qSript XLT cDNA SuperMix (Quanta Biosciences). qPCR was performed using a LightCycler 480 Real‐Time PCR System (Roche). qPCR reactions were duplicated for each sample, using transcript‐specific primers, cDNA (4 ng), and LightCycler 480 SYBR Green I Master (Roche) in a final volume of 10 µL. The PCR data were exported and analyzed in an informatics tool (Gene Expression Analysis Software Environment) developed at the NeuroCentre Magendie. The RefFinder method was used^[^ ^88^ ^]^ to determine the reference gene. Relative expression analysis was normalized against two reference genes, and the succinate dehydrogenase complex subunit and Elongation factor 1‐alpha 1 genes were used. The comparative (2^−∆∆CT^) method^[^ ^89^ ^]^ calculated the relative expression level. Primer sequences are reported in Table S1 (Supporting Information).

### Statistical Analysis

TUSHI data were analyzed in ImageJ Fiji and MS Excel; statistical analysis was done in Graph Pad Prism 9. SPT data (including QD and SWCNT) were analyzed Graph Pad Prism 9. Matrix complexity data were analyzed using Graph Pad Prism 9. qPCR analysis and statistics were performed using the GEASE tool (developed by Neurocentre Magendie, Bordeaux, France). Data presentation (e.g., mean ± SD), sample size (*n*) for each statistical analysis, and used statistical methods were specified in each of the figure legends.

## Conflict of Interest

E.B. is the Chief Scientific Officer of Motac Neuroscience Ltd. All other authors declare no competing interests.

## Author Contributions

J.E.‐P., Y.D., I.C., U.V.N., E.B. contributed equally to this work. Conceptualization: J.E.‐P., Y.D., I.C., U.V.N., E.B. Methodology: J.E.‐P., Y.D., I.C., U.V.N., E.B. Investigation: J.E.‐P., Y.D., I.C., S.N., Q.G., E.D., T.L.‐L. Resources: T.A. Supervision: L.C., L.G., U.V.N., E.B. Writing—original draft: J.E.‐P., Y.D., I.C., U.V.N., E.B. Writing—review and editing: all authors contributed and revised the final version.

## Supporting information



Supporting Information

Supplemental Video 1

Supplemental Video 2

Supplemental Video 3

Supplemental Video 4

Supplemental Video 5

Supplemental Video 6

Supplemental Video 7

Supplemental Video 8

Supplemental Video 9

## Data Availability

The data that support the findings of this study are openly available in [Zenodo] at [https://doi.org/10.5281/zenodo.15056592], reference number [15056592]. The source data for all graphs and charts are available at https://doi.org/10.5281/zenodo.15056592 (DOI). Customs scripts and software code generated for this paper are available from the corresponding authors upon reasonable request.
